# Supplementary material for: Mapping the evolution of fertility support policies in China: A content and instrumental analysis
Source: PLoS One. 2025 Oct 9;20(10):e0332137. doi: 10.1371/journal.pone.0332137 (PMC12510515; doi:10.1371/journal.pone.0332137)
Supplement: S1 Appendix — (ZIP) [file pone.0332137.s001.zip › S1 Appendix. 226 original policy documents/84-国务院关于印发中国妇女发展纲要和中国儿童发展纲要的通知(2021)(FBM-CLI-2-5077239).doc]

国务院关于印发中国妇女发展纲要和中国儿童发展纲要的通知(2021)

**发布部门：** [国务院](https://www.pkulaw.com/law/chl?Aggs.IssueDepartment=5)

**发文字号：**国发〔2021〕16号

**发布日期：**2021.09.08

**实施日期：**2021.09.08

**时效性：** [现行有效](https://www.pkulaw.com/law/chl?Aggs.TimelinessDic=01)

**效力级别：** [国务院规范性文件](https://www.pkulaw.com/law/chl?Aggs.EffectivenessDic=XC0203)

**法规类别：** [老少妇幼残保护](https://www.pkulaw.com/law/chl?Aggs.Category=01604)

**专题分类：** [扫黑除恶](https://www.pkulaw.com/law/chl?Aggs.SpecialType=004)

国务院关于印发中国妇女发展纲要和中国儿童发展纲要的通知
（国发〔2021〕16号）

各省、自治区、直辖市人民政府，国务院各部委、各直属机构：
　　现将《[中国妇女发展纲要（2021-2030年）](https://www.pkulaw.com/chl/37733dd7246afab4bdfb.html)》和《中国儿童发展纲要（2021-2030年）》印发给你们，请认真贯彻执行。

国务院
　　2021年9月8日

[中国妇女发展纲要](https://www.pkulaw.com/chl/37733dd7246afab4bdfb.html)（2021-2030年）
前　　言

　　追求男女平等的事业是伟大的。妇女是人类文明的开创者、社会进步的推动者，是全面建设社会主义现代化国家的重要力量。男女平等和妇女全面发展程度，是衡量社会文明进步的重要标志。党和国家高度重视妇女事业发展，先后制定实施了三个周期的[中国妇女发展纲要](https://www.pkulaw.com/chl/37733dd7246afab4bdfb.html)，为优化妇女发展环境、保障妇女合法权益提供了重要保障。
　　党的十八大以来，以习近平同志为核心的党中央将“坚持男女平等基本国策，保障妇女儿童合法权益”写入党的施政纲领，作为治国理政的重要内容，不断完善党委领导、政府主责、妇女儿童工作委员会（以下简称妇儿工委）协调、多部门合作、全社会参与的妇女工作机制，在出台法律、制定政策、编制规划、部署工作时充分考虑两性的现实差异和妇女的特殊利益，支持妇女充分发挥“半边天”作用，为促进妇女全面发展加速行动。妇女参与经济社会发展的能力和贡献率明显提升，社会地位显著提高，合法权益得到有效保障，健康状况得到极大改善，受教育程度不断提高，参与决策和管理的途径更加多元，社会保障水平稳步提升，在家庭生活中的重要作用进一步彰显，发展环境日益优化。妇女事业和男女平等发展取得了历史性新成就。
　　进入新时代，我国社会主要矛盾发生历史性变化，妇女群众对美好生活的需要日益广泛，妇女发展的不平衡不充分问题仍然突出。城乡、区域和群体之间妇女发展存在差距，农村特别是欠发达地区妇女民生保障力度还需加大。妇女在就业、人身财产、婚姻家庭等方面平等权利的保障仍面临现实困难。妇女参与国家和经济文化社会事务管理的水平有待全面提升。针对妇女各种形式的歧视不同程度存在，性别平等观念有待进一步普及，妇女发展的社会环境需要进一步优化。让性别平等落到实处、推动妇女走在时代前列，使命艰巨、任重道远。
　　当前，我国正处于实现“两个一百年”奋斗目标的历史交汇期。统筹推进“五位一体”总体布局，协调推进“四个全面”战略布局，推进国家治理体系和治理能力现代化，为更高水平促进男女平等和妇女全面发展提供了重大机遇。贯彻以人民为中心的发展思想，坚持新发展理念，坚持系统观念，对充分发挥妇女在社会生活和家庭生活中的独特作用，显著增强妇女的获得感、幸福感、安全感提出了更高要求。世界百年未有之大变局加速演进，推动构建人类命运共同体，建设一个妇女免于歧视的世界，打造一个包容发展的社会，对推动全球性别平等事业发展提出了新的要求。必须在把握新发展阶段、贯彻新发展理念、构建新发展格局中，科学规划妇女全面发展的新目标新任务，健全完善制度机制，团结引领妇女建功新时代、奋进新征程。
　　依照[宪法](https://www.pkulaw.com/chl/7c7e81f43957c58bbdfb.html)和[民法典](https://www.pkulaw.com/chl/aa00daaeb5a4fe4ebdfb.html)、[妇女权益保障法](https://www.pkulaw.com/chl/f20e7cd055022d39bdfb.html)等有关法律法规，按照国家经济社会发展的总体目标要求以及男女平等和妇女发展实际，参照联合国《[消除对妇女一切形式歧视公约](https://www.pkulaw.com/eagn/6e4fcae45b66fcb61dbedc24adbd033dbdfb.html)》和2030年可持续发展议程等国际公约和文件宗旨，制定本纲要。
　　**一、**指导思想、基本原则和总体目标
　　（一）指导思想。
　　高举中国特色社会主义伟大旗帜，深入贯彻党的十九大和十九届二中、三中、四中、五中全会精神，坚持以马克思列宁主义、毛泽东思想、邓小平理论、“三个代表”重要思想、科学发展观、习近平新时代中国特色社会主义思想为指导，坚定不移贯彻新发展理念，坚持以人民为中心的发展思想，坚持走中国特色社会主义妇女发展道路，贯彻落实男女平等基本国策，不断完善促进男女平等和妇女全面发展的制度机制，推动性别平等成为全社会共同遵循的行为规范和价值标准，充分发挥妇女在全面建设社会主义现代化国家中的“半边天”作用，保障妇女平等依法行使民主权利、平等参与经济社会发展、平等享有改革发展成果，推动妇女走在时代前列。
　　（二）基本原则。
　　1．坚持党的全面领导。坚持妇女事业发展的正确政治方向，贯彻落实党中央关于妇女事业发展的决策部署，切实把党的领导贯穿到妇女事业发展的全过程和各方面。
　　2．坚持妇女事业与经济社会同步协调发展。将促进妇女全面发展目标任务纳入国家和地方经济社会发展总体规划，纳入专项规划，纳入民生实事项目，同部署、同落实，让经济社会发展成果更多更公平惠及广大妇女。
　　3．坚持男女两性平等发展。贯彻落实男女平等基本国策，在出台法律、制定政策、编制规划、部署工作时充分考虑两性的现实差异和妇女的特殊利益，营造更加平等、包容、可持续的发展环境，缩小男女两性发展差距。
　　4．坚持促进妇女全面发展。统筹兼顾妇女在政治、经济、文化、社会和家庭各方面的发展利益，有效解决制约妇女发展的重点难点问题，统筹推进城乡、区域、群体之间妇女的均衡发展，协调推进妇女在各领域的全面发展。
　　5．坚持共建共治共享。在统筹推进“五位一体”总体布局、协调推进“四个全面”战略布局中充分发挥妇女的重要作用，促进妇女积极投身高质量发展，踊跃参与国家治理体系和治理能力现代化进程，共享经济社会发展成果。
　　（三）总体目标。
　　男女平等基本国策得到深入贯彻落实，促进男女平等和妇女全面发展的制度机制创新完善。妇女平等享有全方位全生命周期健康服务，健康水平持续提升。妇女平等享有受教育权利，素质能力持续提高。妇女平等享有经济权益，经济地位稳步提升。妇女平等享有政治权利，参与国家和经济文化社会事务管理的水平逐步提高。妇女平等享有多层次可持续的社会保障，待遇水平稳步提高。支持家庭发展的法规政策体系更加完善，社会主义家庭文明新风尚广泛弘扬。男女平等理念更加深入人心，妇女发展环境更为优化。法治体系更加健全，妇女合法权益得到切实保障。妇女的获得感、幸福感、安全感显著提升。展望2035年，与国家基本实现社会主义现代化相适应，男女平等和妇女全面发展取得更为明显的实质性进展，妇女更好地担负起新时代赋予的光荣使命，为实现中华民族伟大复兴的中国梦而不懈奋斗。

　　**二、**发展领域、主要目标和策略措施
　　（一）妇女与健康。
　　主要目标：
　　1．妇女全生命周期享有良好的卫生健康服务，妇女人均预期寿命延长，人均健康预期寿命提高。
　　2．孕产妇死亡率下降到12/10万以下，城乡、区域差距缩小。
　　3．妇女的宫颈癌和乳腺癌防治意识明显提高。宫颈癌和乳腺癌综合防治能力不断增强。适龄妇女宫颈癌人群筛查率达到70%以上，乳腺癌人群筛查率逐步提高。
　　4．生殖健康和优生优育知识全面普及，促进健康孕育，减少非意愿妊娠。
　　5．减少艾滋病、梅毒和乙肝母婴传播，艾滋病母婴传播率下降到2%以下。
　　6．妇女心理健康素养水平不断提升。妇女焦虑障碍、抑郁症患病率上升趋势减缓。
　　7．普及健康知识，提高妇女健康素养水平。
　　8．改善妇女营养状况。预防和减少孕产妇贫血。
　　9．提高妇女经常参加体育锻炼的人数比例，提高妇女体质测定标准合格比例。
　　10．健全妇幼健康服务体系，提升妇幼健康服务能力，妇女健康水平不断提高。
　　策略措施：
　　1．完善保障妇女健康的制度机制。全面推进健康中国建设，把保障人民健康放在优先发展的战略位置，坚持预防为主，深入实施“健康中国行动”和“健康中国母亲行动”，健全政府主导、部门协同、社会参与、行业监管、科技支撑的妇女健康保障工作机制。深入推进医疗、医保、医药联动改革，统筹改革监管体制，保障妇女获得高质量、有效率、可负担的医疗和保健服务。多渠道支持妇女健康事业发展。完善公共卫生应急管理体系，关注妇女的特殊需求。
　　2．加强妇幼健康服务体系建设。健全以妇幼保健机构为核心、以基层医疗卫生机构为基础、以大中型医院和教学科研机构为支撑的妇幼健康服务网络，提升妇幼健康服务供给能力和水平。省、市、县级充分利用现有资源，加强政府举办、标准化的妇幼保健机构建设，全面开展妇幼保健机构绩效考核，强化考核结果应用，保障妇女儿童享有高质量的医疗保健服务。省、市、县级依托现有医疗机构，全面加强危重孕产妇救治中心建设，强化危重孕产妇救治保障。强化县、乡、村三级妇幼卫生服务网络建设，完善基层网底和转诊网络。加强复合型妇幼健康人才和产科、助产等岗位急需紧缺人才的培养使用。
　　3．建立完善妇女全生命周期健康管理模式。针对青春期、育龄期、孕产期、更年期和老年期妇女的健康需求，提供全方位健康管理服务。坚持保健与临床结合，预防为主、关口前移，发挥多学科协作优势，积极发挥中医药在妇幼保健和疾病防治中的作用。为妇女提供宣传教育、咨询指导、筛查评估、综合干预和应急救治等全方位卫生健康服务，提高妇女健康水平和人均健康预期寿命。加强监管，促进妇幼健康新业态规范发展。
　　4．保障孕产妇安全分娩。提倡科学备孕和适龄怀孕，保持适宜生育间隔，合理控制剖宫产率。完善医疗机构产科质量规范化管理体系。提供生育全程基本医疗保健服务，将孕产妇健康管理纳入基本公共卫生服务范围，孕产妇系统管理率达到90%以上。加强对流动孕产妇的管理服务。为低收入孕产妇住院分娩和危重孕产妇救治提供必要救助。持续推进高龄孕产妇等重点人群的分类管理和服务。全面落实妊娠风险筛查与评估、高危孕产妇专案管理、危急重症救治、孕产妇死亡个案报告和约谈通报制度。有效运行危重孕产妇救治网络，提高危急重症救治能力。
　　5．完善宫颈癌和乳腺癌综合防治体系和救助政策。提高妇女的宫颈癌和乳腺癌防治意识和能力，宫颈癌和乳腺癌防治知识知晓率达到90%以上。推进适龄妇女人乳头瘤病毒疫苗接种试点工作。落实基本公共卫生服务中农村妇女宫颈癌和乳腺癌检查项目，促进70%的妇女在35-45岁接受高效宫颈癌筛查，督促用人单位落实[女职工保健工作规定](https://www.pkulaw.com/chl/6bd61140293005a1bdfb.html)，定期进行女职工宫颈癌和乳腺癌筛查，提高人群筛查率。加强宫颈癌和乳腺癌筛查和诊断技术创新应用，提高筛查和服务能力，加强监测评估。强化筛查和后续诊治服务的衔接，促进早诊早治，宫颈癌患者治疗率达到90%以上。加强对困难患者的救助。
　　6．提高妇女生殖健康水平。普及生殖道感染、性传播疾病等疾病防控知识。在学校教育不同阶段以多种形式开展科学、实用的健康教育，促进学生掌握生殖健康知识，提高自我保护能力。增强男女两性性道德、性健康、性安全意识，倡导共担避孕责任。将生殖健康服务融入妇女健康管理全过程，保障妇女享有避孕节育知情自主选择权。落实基本避孕服务项目，加强产后和流产后避孕节育服务，提高服务可及性，预防非意愿妊娠。推进婚前医学检查、孕前优生健康检查、增补叶酸等婚前孕前保健服务更加公平可及。减少非医学需要的人工流产。加强对女性健康安全用品产品的质量保障。规范不孕不育症诊疗服务。规范人类辅助生殖技术应用。
　　7．加强艾滋病梅毒乙肝母婴传播防治。全面落实预防艾滋病、梅毒和乙肝母婴传播综合干预措施，提高孕早期检测率，孕产妇艾滋病、梅毒和乙肝检测率达到98%以上，艾滋病、梅毒孕产妇感染者治疗率达到95%以上。加大艾滋病防控力度，加强艾滋病防治知识和相关政策宣传教育，提高妇女的防范意识和能力。加强对妇女感染者特别是流动和欠发达地区妇女感染者的医疗服务，提高随访率。为孕产妇感染者及其家庭提供多种形式的健康咨询、心理和社会支持等服务。
　　8．促进妇女心理健康。加强心理健康相关知识宣传，根据妇女需要开展心理咨询、评估和指导，促进妇女掌握基本的心理调适方法，预防抑郁、焦虑等心理问题。在心理健康和精神卫生服务体系建设中，重点关注青春期、孕产期、更年期和老年期妇女的心理健康。强化心理咨询和治疗技术在妇女保健和疾病防治中的应用。加大应用型心理健康和社会工作人员培养力度，促进医疗机构、心理健康和社会工作服务机构提供规范服务。鼓励社区为有需要的妇女提供心理健康服务支持。
　　9．提升妇女健康素养。实施健康知识普及行动，加大妇女健康知识普及力度，建立完善健康科普专家库和资源库，持续深入开展健康科普宣传教育，规范发布妇女健康信息，引导妇女树立科学的健康理念，学习健康知识，掌握身心健康、预防疾病、科学就医、合理用药等知识技能。提高妇女参与传染病防控、应急避险的意识和能力。面向妇女开展控制烟草危害、拒绝酗酒、远离毒品宣传教育。引导妇女积极投身爱国卫生运动，养成文明健康生活方式。
　　10．提高妇女营养水平。持续开展营养健康科普宣传教育，因地制宜开展营养和膳食指导，提高妇女对营养标签的知晓率，促进妇女学习掌握营养知识，均衡饮食、吃动平衡，预防控制营养不良和肥胖。面向不同年龄阶段妇女群体开发营养健康宣传信息和产品，提供有针对性的服务。开展孕产妇营养监测和定期评估，预防和减少孕产妇缺铁性贫血。预防控制老年妇女低体重和贫血。
　　11．引导妇女积极参与全民健身行动。完善全民健身公共服务体系。引导妇女有效利用全民健身场地设施，积极参与全民健身赛事活动，加入各类健身组织。提倡机关、企事业单位开展工间操。鼓励支持工会组织、社区开展妇女健身活动，不断提高妇女的体育活动意识，培养运动习惯。
　　12．强化妇女健康服务科技支撑。推进“互联网＋妇幼健康”，促进大数据、云计算、人工智能、计算机仿真技术等在妇女健康领域的创新应用。实施妇女人群健康管理和健康风险预警。促进信息技术在妇女健康领域专科医联体建设中的应用，加强医疗机构间的协作，促进分级诊疗和上下联动。促进妇女身心健康领域的科学研究和成果转化。发挥妇产疾病领域国家临床医学研究中心的作用。
　　（二）妇女与教育。
　　主要目标：
　　1．加强思想政治教育，增进妇女对习近平新时代中国特色社会主义思想的政治认同、思想认同、情感认同，引领妇女做伟大事业的建设者、文明风尚的倡导者、敢于追梦的奋斗者。
　　2．教育工作全面贯彻男女平等基本国策。
　　3．大中小学性别平等教育全面推进，教师和学生的男女平等意识明显增强。
　　4．女童平等接受义务教育，九年义务教育巩固率提高到96%以上。
　　5．女性平等接受高中阶段教育，高中阶段教育毛入学率达到并保持在92%以上。
　　6．女性接受职业教育的水平逐步提高。
　　7．高校在校生中男女比例保持均衡，高等教育学科专业的性别结构逐步趋于平衡。
　　8．大力培养女性科技人才。男女两性的科学素质水平差距不断缩小。
　　9．促进女性树立终身学习意识，女性接受终身教育水平不断提高。
　　10．女性青壮年文盲基本消除。女性平均受教育年限不断提高。
　　策略措施：
　　1．面向妇女广泛开展思想政治教育。深入开展习近平新时代中国特色社会主义思想学习教育，加强党史、新中国史、改革开放史、社会主义发展史教育，加强爱国主义、集体主义、社会主义教育，促进妇女更加坚定理想信念，不断厚植爱国情怀，把个人理想追求融入党和国家事业大局，为全面建设社会主义现代化国家贡献力量。深化民族团结进步教育，铸牢中华民族共同体意识。充分发挥学校教育主阵地作用，将思想价值引领贯穿于教育教学及管理全过程和校园生活各方面，融入学校党组织、共青团、少先队各类主题教育和实践活动。充分发挥爱国主义教育基地和国防教育基地的思想政治教育作用。
　　2．将贯彻落实男女平等基本国策体现在教育工作全过程。增强教育工作者自觉贯彻男女平等基本国策的主动性和能动性。将男女平等基本国策落实到教育法规政策和规划制定、修订、执行和评估中，落实到各级各类教育内容、教学过程、学校管理中。加强对教材编制、课程设置、教学过程的性别平等评估。在师范类院校课程设置和教学、各级各类师资培训中加入性别平等内容。
　　3．推动各级各类学校广泛开展性别平等教育。适时出台性别平等教育工作指导意见。推动因地制宜开发性别平等课程，加强专题师资培训。促进性别平等教育融入学校教学内容、校园文化、社团活动和社会实践活动。探索构建学校教育、家庭教育、社会教育相结合的性别平等教育模式。
　　4．保障女童平等接受义务教育的权利和机会。深化教育教学改革，加快城乡义务教育一体化发展，均衡配置教育资源，确保女童平等接受公平优质的义务教育。健全精准控辍保学长效机制，加强分类指导，督促法定监护人依法保障女童接受义务教育，切实解决义务教育女童失学辍学问题。保障欠发达地区女童、留守女童、农业转移人口随迁子女以及残疾女童的受教育权利和机会。支持学业困难女童完成义务教育，提高女童义务教育巩固率。
　　5．提高女性接受普通高中教育的比例。保障女性特别是欠发达地区和农村低收入家庭女性平等接受普通高中教育的权利和机会。鼓励普通高中多样化有特色发展，满足女性全面发展和个性化发展需求。有针对性地开展学科选择和职业生涯规划指导，提高女性自主选择能力，破除性别因素对女性学业和职业发展的影响。
　　6．促进女性接受高质量职业教育。完善学历教育与培训并重的现代职业教育体系，优化专业设置，提供多种学习方式，支持女性获得职业技能等级证书，培养复合型技术技能女性人才和能工巧匠、大国工匠。鼓励职业院校面向高校女毕业生、女农民工、去产能分流女职工等重点人群开展就业创业和职业技能培训。
　　7．保障女性平等接受高等教育的权利和机会。严格控制招生过程中的特殊专业范围，强化监管，建立约谈、处罚机制。保持高校在校生中男女比例的均衡。采取激励措施，提高女性在科学、技术、工程、数学等学科学生中的比例，支持数理化生等基础学科基地和前沿科学中心建设，加强对基础学科拔尖女生的培养。
　　8．大力提高女性科学素质。开展全民科学素质行动，利用现代信息化手段，加大面向女性的科学知识教育、传播与普及力度。开展女科学家进校园活动，发挥优秀女科技人才的榜样引领作用。引导中小学女生参加各类科普活动和科技竞赛，培养科学兴趣、创新精神和实践能力。鼓励女大学生积极参与项目设计、社会实践、创新创业、科技竞赛等活动。深入实施农村妇女素质提升计划，支持农村妇女参与农业农村现代化建设。
　　9．大力加强女性科技人才培养。探索建立多层次女性科技人才培养体系，培养具有国际竞争力的女性科技人才。关注培养义务教育阶段女生爱科学、学科学的兴趣和志向。引导高中阶段女生养成科学兴趣和钻研精神，支持有意愿的女生报考理工类院校。加大女性创新型、应用型人才培养力度，鼓励女大学生参与科研项目，在实践中培养科学精神和创新能力。引导女性从事科学和技术相关工作，增加女性科技人才参与继续教育和专业培训的机会。
　　10．为女性终身学习提供支持。建立完善更加开放灵活的终身学习体系，完善注册学习、弹性学习和继续教育制度，拓宽学历教育渠道，满足女性多样化学习需求，关注因生育中断学业和职业女性的发展需求。建立健全国家学分银行和学习成果认定制度。扩大教育资源供给，为女性提供便捷的社区和在线教育，为进城务工女性、女性新市民、待业女性等提供有针对性的职业技能培训。
　　11．持续巩固女性青壮年扫盲成果，加大普通话推广力度。完善扫盲工作机制，加强国家通用语言文字教育，消除女童辍学现象，杜绝产生女性青壮年新文盲。普通话培训及各类职业培训向欠发达地区妇女和残疾妇女等群体倾斜。深化扫盲后的继续教育。提高妇女平均受教育年限。
　　12．加强女性学研究和人才培养。加强女子高校建设，推动有条件的高校开设妇女研究及性别平等相关课程。培养具有跨学科知识基础和性别平等意识的专业人才。加大对妇女理论研究的支持力度，加强跨学科研究，提高国家社科基金项目等重大研究项目中妇女或性别研究相关选题的立项比例。
　　13．构建平等尊重和安全友善的校园环境。促进建立相互尊重、平等和睦的师生、同学关系，鼓励学校设置生命教育、心理健康教育和防性侵、防性骚扰的相关课程，提高学生的自我保护意识和能力。中小学校建立完善预防性侵未成年人工作机制，高校建立完善预防性侵和性骚扰工作机制，加强日常管理、预防排查、投诉受理和调查处置。加强师德师风建设，履行查询法定义务，对不符合条件的教职人员进行处置。
　　（三）妇女与经济。
　　主要目标：
　　1．鼓励支持妇女为推动经济高质量发展贡献力量，妇女平等参与经济发展的权利和机会得到保障。
　　2．促进平等就业，消除就业性别歧视。就业人员中的女性比例保持在45%左右。促进女大学生充分就业。
　　3．优化妇女就业结构。城镇单位就业人员中的女性比例达到40%左右。
　　4．促进女性人才发展。高级专业技术人员中的女性比例达到40%，促进女性劳动者提升职业技能水平。
　　5．保障妇女获得公平的劳动报酬，男女收入差距明显缩小。
　　6．保障女性劳动者劳动安全和健康。女职工职业病发病率明显降低。
　　7．保障农村妇女平等享有土地承包经营权、宅基地使用权等权益，平等享有农村集体经济组织收益分配、土地征收或征用安置补偿权益。
　　8．巩固拓展脱贫攻坚成果，增强农村低收入妇女群体的可持续发展能力。
　　9．妇女在实施乡村振兴战略中的作用充分发挥。
　　策略措施：
　　1．完善保障妇女平等获得经济资源、参与经济建设、享有经济发展成果的法律法规政策。制定实施支持女性科技人才在创新发展中发挥更大作用的政策措施。创新制度机制，保障妇女在就业创业、职业发展、劳动报酬、职业健康与安全、职业退出、土地等方面的权益，保障新业态从业人员劳动权益，为妇女充分参与经济高质量发展创造有利条件。
　　2．加大消除就业性别歧视工作力度。全面落实消除就业性别歧视的法律法规政策，创造性别平等的就业机制和市场环境。对招聘、录用环节涉嫌性别歧视的用人单位进行联合约谈，依法惩处。督促用人单位加强就业性别歧视自查自纠。发挥劳动保障法律监督作用，对涉嫌就业性别歧视的用人单位提出纠正意见，或者向相关行政部门提出处理建议。依法受理涉及就业性别歧视的诉讼。发挥行业协会、商会协调监督作用，提高行业自律意识。党政机关、国有企事业单位在招录（聘）和职工晋职晋级、评定专业技术职称等方面发挥男女平等的示范引领作用。
　　3．促进妇女就业创业。健全公共就业服务体系，深化就业服务专项活动，促进妇女就业的人岗对接。充分发挥现代服务业和新业态吸纳妇女就业的功能，支持妇女参与新业态新模式从业人员技能培训。加大帮扶力度，多渠道帮助就业困难妇女实现就业。扶持民族传统手工艺品产业发展，提高组织化程度，促进各族妇女就地就近就业。支持女性科技人才投身科技创业，发展农村电子商务，鼓励外出务工妇女返乡创业，支持有意愿的妇女下乡创业。创新金融、保险产品和服务模式，拓宽妇女创业融资渠道。
　　4．促进女大学生就业创业。加强职业生涯规划指导服务，引导女大学生树立正确的择业就业观，提升就业能力。完善落实就业创业支持政策，高校和属地政府提供不断线的就业服务，拓宽女大学生市场化社会化就业渠道。鼓励女大学生到基层、中小微企业或新经济领域就业。推广女大学生创业导师制，开展女大学生创新创业大赛，支持女大学生创业。对有就业意愿的离校未就业女毕业生提供就业帮扶。
　　5．改善妇女就业结构。完善终身职业技能培训制度，提升妇女职业技能水平，大力培育知识型、技能型、创新型女性劳动者。不断提高妇女在高新技术产业、战略性新兴产业和现代服务业从业人员中的比例。逐步消除职业性别隔离，提高城镇单位就业人员中的女性比例。扩大农村妇女转移就业规模，缩小男女转移就业差距。
　　6．加强女性专业技术和技能人才队伍建设。制定相关政策，强化制度保障，支持女性科技人才承担科技计划项目、参与科技决策咨询、拓展科研学术网络、提升国际影响力和活跃度，完善女性科技人才评价激励机制，培养高层次女性科技人才。实施科技创新巾帼行动，搭建平台、提供服务，激励女性科技人才、技术技能人才立足岗位锐意创新。加强对女性专业技术和技能人才专业知识、科研管理、创新创业等的培训。加强典型宣传，发挥榜样引领作用。
　　7．缩小男女两性收入差距。全面落实男女同工同酬，保障收入公平。促进女性对知识、技术、管理、数据等生产要素的掌握和应用，提高女性职业竞争力。督促用人单位制定实施男女平等的人力资源制度，畅通女性职业发展和职务职级晋升通道。探索开展薪酬调查，加强对收入的分性别统计，动态掌握男女两性收入状况。
　　8．改善女性劳动者劳动安全状况。广泛开展劳动安全和健康宣传教育，加大《[女职工劳动保护特别规定](https://www.pkulaw.com/chl/cdf82292dbb62ee9bdfb.html)》宣传执行力度，提高用人单位和女性劳动者的劳动保护和安全生产意识。将女职工劳动保护纳入职业健康和安全生产监督管理范围，加强对用人单位的劳动保障监察以及劳动安全和职业健康监督。督促用人单位加强对女职工经期、孕期、哺乳期的特殊保护，落实哺乳时间和产假制度。督促用人单位加强职业防护和职业健康监督保护，保障女职工在工作中免受有毒有害物质和危险生产工艺的危害。
　　9．保障女职工劳动权益。督促用人单位规范用工行为，依法与女职工签订劳动合同，推动签订女职工权益保护专项集体合同。加强劳动保障法律监督。指导用人单位建立预防和制止性骚扰工作机制，完善相关执法措施。加强劳动用工领域信用建设，加大对侵犯女职工劳动权益行为的失信惩戒力度。推动有条件的劳动人事争议仲裁机构设立女职工维权仲裁庭，依法处理女职工劳动争议案件。
　　10．为女性生育后的职业发展创造有利条件。禁止用人单位因女职工怀孕、生育、哺乳而降低工资、恶意调岗、予以辞退、解除劳动（聘用）合同，推动落实生育奖励假期间的工资待遇，定期开展女职工生育权益保障专项督查。为女性生育后回归岗位或再就业提供培训等支持。高校、研究机构等用人单位探索设立女性科研人员生育后科研回归基金。推动用人单位根据女职工需要建立女职工哺乳室、孕妇休息室等设施。支持有条件的用人单位为职工提供福利性托育托管服务。
　　11．保障农村妇女平等享有各项经济权益。在农村土地承包工作中，依法保障农村妇女权益。在宅基地使用权确权登记颁证工作中保障农村妇女权益，确保应登尽登。建立健全农村集体资产管理制度，规范农村集体经济组织成员身份确认办法，完善包括征地补偿安置在内的农村集体经济组织资产收益内部分配机制，保障妇女在农村集体经济组织资产股权量化、权益流转和继承等各环节，作为农村集体经济组织成员和家庭成员平等享有知情权、参与决策权和收益权。保障进城落户女农民的经济权益。畅通经济权益受侵害农村妇女的维权渠道。
　　12．支持脱贫妇女稳定增加收入。建立农村低收入人口和欠发达地区帮扶机制。健全防止返贫监测和帮扶机制。扶持发展适合城乡低收入妇女自主发展的手工编织、农村电商等特色产业项目。通过致富带头人培育、帮扶车间建设和以工代赈等方式，支持农村妇女就地就近就业、实现增收致富。
　　13．支持妇女积极参与乡村振兴。积极发挥妇女在农村一二三产业融合发展和农业农村现代化建设中的作用。大力开展现代农业示范基地建设，深入实施乡村振兴巾帼行动。发挥农村创业创新园区（基地）等平台作用，鼓励支持妇女创办领办新型农业经营主体和农业社会化服务组织。加强高素质女农民培育，引导女农民争做乡村工匠、文化能人、手工艺人、农技协领办人和新型农业经营管理能手。
　　（四）妇女参与决策和管理。
　　主要目标：
　　1．保障妇女参与社会主义民主政治建设和社会治理，提升参与水平。
　　2．中国共产党女党员保持合理比例。中国共产党各级党员代表大会中女党员代表比例一般不低于本地区党员总数中女性比例。
　　3．各级人大代表和常委会委员中的女性比例逐步提高。各级政协委员和常委中的女性比例逐步提高。
　　4．县级以上地方政府领导班子中的女干部比例逐步提高，担任正职的女干部占同级正职干部的比例逐步提高。
　　5．国家机关部委和县级以上地方政府部门领导班子中的女干部比例逐步提高，担任正职的女干部占同级正职干部的比例逐步提高。
　　6．各级各类事业单位领导班子成员中的女性比例逐步提高。
　　7．企业董事会、监事会成员及管理层中的女性比例逐步提高。企事业单位职工代表大会中女性比例与女职工比例相适应。
　　8．村党组织成员、村党组织书记中女性比例逐步提高。村委会成员中女性比例达到30%以上，村委会主任中女性比例逐步提高。
　　9．社区党组织成员、社区党组织书记中女性比例逐步提高。社区居委会成员中女性比例保持在50%左右，社区居委会主任中女性比例达到40%以上。
　　10．鼓励支持女性参加社会组织、担任社会组织负责人。
　　策略措施：
　　1．加大对妇女参与决策和管理的支持力度。充分发挥妇女参与国家和社会事务管理的重要作用，破除制约妇女参与决策和管理的障碍，促进妇女参与决策和管理水平与妇女地位作用相适应。加大培训力度，提高各级领导干部贯彻落实男女平等基本国策的意识，把推动妇女参政纳入重要议程，提出目标举措。采取有效措施，提升各级党委、人大、政府、政协、党政工作部门以及企事业单位、基层群众自治组织和社会组织中的女性比例。
　　2．提高妇女参与社会事务和民主管理的意识和能力。开展女性领导干部政治素质和领导能力培训。鼓励高校开设领导力相关课程，培养年轻女性的政治素养及参与决策和管理的意识。加大基层妇女骨干培训力度，提高妇女在自治、法治、德治中的参与意识和能力，鼓励妇女积极参与村（居）民议事会、理事会等自治组织，推进城乡社区妇女议事会实现全覆盖并有效运行，发挥妇女在城乡基层治理中的积极作用。探索打造妇女网上议事平台，引导妇女积极、有序参与基层民主管理和基层民主协商。
　　3．重视发展中国共产党女党员。面向妇女深入开展思想政治工作，扩大党的妇女群众基础，培养对党的感情，深化对党的认识，引导拥护党的主张，激发妇女入党的政治意愿。加强对入党积极分子的培养教育。注重从各行各业青年女性中发展党员。在党代表候选人酝酿过程中，充分关注政治过硬、作风优良、敢于担当、实绩突出的优秀妇女，确保党员代表大会中女党员代表保持合理比例。
　　4．提高人大女代表、政协女委员比例。落实人大代表选举规则和程序，在选区划分、代表名额分配、候选人推荐、选举等环节，保障妇女享有平等权利和机会。重视从基层、生产一线推荐人大代表女性候选人，候选人中应当有适当数量的妇女代表，并逐步提高妇女代表的比例。提名推荐、协商确定政协委员建议名单时，保障提名一定比例的妇女。充分发挥人大女代表、政协女委员在发展社会主义民主政治和男女平等事业中的积极作用。
　　5．加大培养选拔女干部工作力度。培养忠诚干净担当的高素质专业化女干部，促进女干部不断增强学习本领、政治领导本领、改革创新本领、科学发展本领、依法执政本领、群众工作本领、狠抓落实本领、驾驭风险本领。优化女干部成长路径，注重日常培养和战略培养，为女干部参加教育培训、交流任职、挂职锻炼创造条件和机会。注重从基层、生产一线培养选拔女干部，注重选拔女干部到重要部门、关键岗位担任领导职务。注重保持优秀年轻干部队伍中女干部的合理比例。落实女干部选拔配备的目标任务，在保证质量的前提下实现应配尽配。保障妇女在干部录用、选拔、任（聘）用、晋升、退休各环节不因性别受到歧视。
　　6．推动妇女积极参与事业单位决策管理。培养选拔优秀女性专业技术人员进入决策管理层。重视在卫生、教育、文化等女性集中的行业提高决策管理层中的女性比例，鼓励妇女积极参与本单位党建和群团组织建设，促进事业单位职工代表大会中的女职工代表比例与事业单位女职工比例相适应。在深化事业单位改革进程中，确保妇女在岗位晋升、职员晋级、职称评聘等方面享有平等的权利和机会。
　　7．推动妇女广泛参与企业决策管理。将女干部选拔配备纳入国有企业领导班子和干部队伍建设规划，加大培养、选拔、使用力度。在深化企业人事制度改革进程中，采用组织推荐、公开招聘、民主推荐等方式，促进优秀妇女进入企业董事会、监事会和管理层。完善企业民主管理制度，促进企业职工代表大会中女职工代表比例与企业女职工比例相适应，支持女职工通过职工代表大会等形式参与企业民主决策、民主管理和民主监督。企业制定相关规章制度，对涉及女职工权益的事项，听取工会女职工委员会的意见，依法经职工代表大会审议通过。
　　8．推动妇女有序参与城乡基层社会治理。注重从女致富能手、经商务工女性、乡村女教师女医生、女社会工作者、女大学生村官、女退休干部职工等群体中培养选拔村（社区）干部。在村（社区）“两委”换届工作中，通过提名确定女性候选人、女性委员专职专选、女性成员缺位增补等措施，提高村（居）委会成员、村（居）委会主任中的女性比例。组织妇女积极参与村规民约、居民公约的制定修订，开展协商议事活动。促进新社会阶层、社会工作者和志愿者中的女性积极参与社会治理。
　　9．支持引导妇女参加社会组织。优化社会组织发展的制度环境，加大对以女性为成员主体或以女性为主要从业人员的社会组织的培育力度，加强支持和指导服务，促进其健康有序发展并积极参与社会组织协商。鼓励支持更多女性成为社会组织成员或从业人员，加强对社会组织女性专业人才和管理人才的培养，注重发现培养社会组织女性负责人。
　　10．发挥妇联组织在推进国家治理体系和治理能力现代化进程中的作用。支持妇联组织履行代表妇女参与管理国家事务、经济文化事业和社会事务的职责，强化妇联组织参与民主决策、民主管理、民主监督，参与制定有关法律、法规、规章和政策，参与社会治理和公共服务的制度保障。在制定有关促进男女平等和保障妇女合法权益的法律法规政策以及培养选拔女干部工作中，充分听取妇联组织意见和建议。
　　（五）妇女与社会保障。
　　主要目标：
　　1．妇女平等享有社会保障权益，保障水平不断提高。
　　2．完善生育保障制度。提高生育保险参保率。
　　3．完善医疗保障体系。妇女基本医疗保险参保率稳定在95%以上，待遇保障公平适度。
　　4．完善养老保险制度体系。妇女基本养老保险参保率提高到95%，待遇水平稳步提高。
　　5．完善失业保险和工伤保险制度。提高妇女失业保险和工伤保险参保人数，落实相关待遇保障。
　　6．健全分层分类社会救助体系。困难妇女的生活得到基本保障。
　　7．妇女福利待遇水平持续提高，重点向老年妇女、残疾妇女等群体倾斜。
　　8．建立完善多层次养老服务和长期照护保障制度。保障老年妇女享有均等可及的基本养老服务，对失能妇女的照护服务水平不断提高。
　　9．加强对妇女的关爱服务，重点为有困难、有需求的妇女提供帮扶。
　　策略措施：
　　1．完善惠及妇女群体的社会保障体系。在制定修订社会救助、社会保险等相关法律法规以及健全覆盖全民的社会保障体系工作中，关切和保障妇女的特殊利益和需求。持续推动社会保险参保扩面，支持灵活就业女性参加相应社会保险，实现应保尽保，缩小社会保障的性别差距。建立国家级社会保险全民参保登记信息库，加强社会保障分性别统计、信息动态监测和管理。
　　2．完善覆盖城乡妇女的生育保障制度。巩固提高生育保险覆盖率，完善生育保险生育医疗费用支付及生育津贴政策。妥善解决妇女在就业和领取失业金期间生育保障问题。提高生育保险与职工基本医疗保险合并实施成效。加强城乡居民生育医疗费用保障。
　　3．不断提高妇女医疗保障水平。推动女职工和城乡女性居民持续参加基本医疗保险，满足妇女基本医疗保障需求。统筹发挥基本医保、大病保险、医疗救助三重制度综合保障作用，促进多层次医疗保障互补衔接，做好符合条件的低收入妇女医疗救助。推进建立女职工医疗互助，充分发挥商业保险对宫颈癌、乳腺癌等重大疾病的保障作用。
　　4．促进妇女享有可持续多层次养老保险。建立完善基本养老保险全国统筹制度。督促用人单位依法为包括女职工在内的全体职工及时足额缴纳基本养老保险费，不断增加妇女参加基本养老保险的人数，促进妇女依法公平享有基本养老保险权益。鼓励有条件的用人单位为包括女职工在内的全体职工建立企业年金，丰富商业养老保险产品，提高妇女养老保险水平。
　　5．保障女性失业保险权益。督促用人单位依法为女职工办理失业保险，提高女职工特别是女农民工的参保率。保障符合条件的失业女职工按时享受失业保险待遇。强化失业保险促就业防失业功能，支持女职工稳定就业。适时制定特殊时期失业保障政策，为包括女职工在内的劳动者提供失业保障。
　　6．扩大妇女工伤保险覆盖面。增强工伤保险预防工伤、保障生活、促进康复的功能，推进新就业形态人员职业伤害保障试点，将新业态就业妇女纳入保障范围。督促用人单位特别是高风险行业单位依法为女职工办理工伤保险，确保落实工伤保险待遇。
　　7．强化社会救助对生活困难妇女的兜底保障。推进法律实施，强化政策衔接，健全基本生活救助制度和医疗救助、教育救助、住房救助、就业救助、受灾人员救助等专项救助制度，健全临时救助政策措施，强化急难社会救助功能，积极发展服务类社会救助，推进政府购买社会救助服务，确保符合条件的妇女应救尽救。鼓励、支持慈善组织依法依规为生活困难妇女提供救助帮扶。推动建立统一的救助信息平台，加强社会救助分性别统计，精准识别救助对象。
　　8．更好满足妇女群体的社会福利需求。完善经济困难高龄失能老年人补贴制度，落实各项补贴待遇，逐步提升老年妇女福利水平。完善残疾人补贴制度，动态调整、合理确定困难残疾人生活补贴和重度残疾人护理补贴标准，扩大适合残疾妇女特殊需求的公共服务供给。
　　9．保障妇女享有基本养老服务。加快建设居家社区机构相协调、医养康养相结合的养老服务体系，大力发展普惠型养老服务。完善社区居家养老服务网络，推进公共设施适老化改造，推动专业机构服务向社区和家庭延伸。提升公办养老机构服务能力和水平，完善公建民营管理机制，结合服务能力适当拓展服务对象，重点为经济困难的失能失智、计划生育特殊家庭老年人提供托养服务。促进养老机构提供多元化、便利化、个性化服务，提高老年妇女生活照料、紧急救援、精神慰藉等服务水平。支持社会力量扩大普惠型养老服务供给，支持邻里之间的互助性养老。加大养老护理型人才培养力度，建设高素质、专业化的养老服务队伍。
　　10．探索建立多层次长期照护保障制度。稳步建立长期护理保险制度，将符合条件的失能妇女按规定纳入保障范围，妥善解决其护理保障问题。加强长期护理保险制度与长期照护服务体系有机衔接。探索建立相关保险、福利、救助相衔接的长期照护保障制度，扩大养老机构护理型床位供给，提高护理服务质量。为家庭照料者提供照护培训、心理疏导等支持。
　　11．提高对妇女的关爱服务水平。开展农村留守妇女关爱行动。对农村留守妇女进行摸底排查，建立完善以县级为单位的信息台账。积极为农村留守妇女创业发展搭建平台、提供服务。支持农村留守妇女参与乡村振兴和家庭文明建设，在乡村治理、邻里互助、留守老人儿童关爱服务中发挥积极作用。完善特殊困难失能留守老年人探访关爱制度，不断拓展对妇女群体的关爱服务，支持社会力量参与，重点为生活困难、残疾、重病等妇女群体提供权益保护、生活帮扶、精神抚慰等关爱服务。
　　（六）妇女与家庭建设。
　　主要目标：
　　1．树立新时代家庭观，弘扬爱国爱家、相亲相爱、向上向善、共建共享的社会主义家庭文明新风尚，推动社会主义核心价值观在家庭落地生根。
　　2．建立完善促进男女平等和妇女全面发展的家庭政策体系，增强家庭功能，提升家庭发展能力。
　　3．拓展支持家庭与妇女全面发展的公共服务。
　　4．注重发挥家庭家教家风在基层社会治理中的重要作用。
　　5．充分发挥妇女在家庭生活中的独特作用，弘扬中华民族家庭美德、树立良好家风，支持妇女成为幸福安康家庭的建设者、倡导者。
　　6．倡导构建男女平等、和睦、文明的婚姻家庭关系，降低婚姻家庭纠纷对妇女发展的不利影响。
　　7．倡导和支持男女共担家务，缩小两性家务劳动时间差距。
　　8．支持家庭承担赡养老人责任，不断提升老年妇女家庭生活质量。
　　9．促进夫妻共同承担未成年子女的抚养、教育、保护责任，为未成年子女身心发展创造良好家庭环境。
　　策略措施：
　　1．促进家庭成员践行社会主义核心价值观。加强教育引导、舆论宣传、文化熏陶、实践养成，宣传尊老爱幼、男女平等、夫妻和睦、勤俭持家、邻里团结等家庭美德，弘扬中华民族优秀传统家风、革命前辈红色家风、践行社会主义核心价值观的现代家风，营造平等、文明、和谐、稳定的家庭环境，实现共建共享的家庭追求，引导妇女和家庭成员自觉把家庭梦融入中国梦。
　　2．制定出台促进男女平等和妇女全面发展的家庭政策。完善人口生育相关法律法规政策，推动生育政策与经济社会政策配套衔接。研究推动将3岁以下婴幼儿照护服务费用纳入个人所得税专项附加扣除、住房等方面支持政策，减轻家庭生育、养育、教育负担。完善幼儿养育、青少年发展、老人赡养、病残照料等政策，形成支持完善家庭基本功能、促进男女平等和妇女全面发展的家庭政策体系，增强家庭发展能力。完善产假制度，探索实施父母育儿假。建立促进家庭发展的政策评估机制。
　　3．大力发展家庭公共服务。发展普惠托育服务体系，综合运用土地、住房、财政、金融、人才等支持政策，扩大托育服务供给。加快完善养老、家政等服务标准，推动婚姻家庭辅导服务、家庭教育指导服务普惠享有，提升面向家庭的公共服务水平。通过政府购买服务等方式，引导社会力量开展家庭服务，满足家庭日益增长的个性化、多元化需求。重点为经济困难、住房困难、临时遭遇困难家庭和残疾人家庭等提供支持，加大对计划生育特殊家庭的帮扶保障力度，加强对退役军人家庭的支持和保障。城市社区综合服务设施实现全覆盖。加强社区托育服务设施建设，完善社区养老托育、家政物业等服务网络。发展数字家庭。
　　4．推动家庭家教家风在基层社会治理中发挥重要作用。构建党委领导、政府主导、部门合作、家庭尽责、社会参与的家庭建设工作格局。将建设好家庭、实施好家教、弘扬好家风纳入基层社会治理体系以及基层社会治理评价考核内容。鼓励家庭成员履行家庭和社会责任。增进政府治理和社会调节、居民自治良性互动，以千千万万家庭的好家风支撑起全社会的好风气。
　　5．鼓励支持妇女在家庭生活中发挥独特作用。深化实施“家家幸福安康工程”，鼓励妇女带领家庭成员积极参与文明家庭、五好家庭、最美家庭等群众性精神文明建设活动，参与绿色家庭创建，提升健康素养，践行绿色、低碳、循环、可持续的生活方式，养成勤俭节约的好习惯，杜绝浪费。推进平安家庭、无烟家庭建设。
　　6．促进婚姻家庭关系健康发展。面向家庭开展有关法律法规政策宣传，促进男女平等观念在婚姻家庭关系建设中落实落地，倡导夫妻平等参与家庭事务决策，反对一切形式的家庭暴力。开展恋爱、婚姻家庭观念教育，为适龄男女青年婚恋交友、组建家庭搭建平台，推广婚姻登记、婚育健康宣传教育、婚姻家庭关系辅导等“一站式”服务。广泛开展生育政策宣传。推进移风易俗，保障各民族妇女的婚姻自由，抵制早婚早育、高价彩礼等现象，选树宣传婚事新办典型，引导改变生男偏好，构建新型婚育文化。加强对广播电视、网络等婚恋活动和服务的规范管理。
　　7．加强婚姻家庭纠纷预防化解工作。健全婚姻家庭纠纷预防化解工作机制，发挥综治中心和网格化服务管理作用，强化衔接联动，加强婚姻家庭纠纷预测预防预警，健全纠纷排查调处制度。推进县（市、区、旗）建立健全婚姻家庭纠纷人民调解委员会，建设人民调解员队伍，搭建“互联网＋”纠纷预防化解工作平台，支持社会力量参与，提供多元便捷服务。推进家事审判制度改革，加强诉调对接平台建设，构建新型家事纠纷综合协调解决模式。
　　8．促进男女平等分担家务。倡导夫妻在家务劳动中分工配合，共同承担照料陪伴子女老人、教育子女、料理家务等家庭责任，缩小两性家务劳动时间差距。促进照料、保洁、烹饪等家务劳动社会化，持续推动家政服务业提质扩容增效，发展婴幼儿照护服务和失能失智老年人长期照护服务，增强家庭照护能力，研发家务劳动便利化产品。督促用人单位落实探亲假、职工带薪休假、配偶陪产假等制度，鼓励用人单位实施灵活休假和弹性工作制度，创造生育友好的工作环境，支持男女职工共同履行家庭责任。
　　9．提高老年妇女的家庭生活质量。倡导养老、孝老、敬老的家庭美德，支持家庭履行赡养老人的主体责任。鼓励子女与老年人共同生活或就近居住，为长期照护老年人的家庭成员提供“喘息服务”。督促用人单位保障赡养义务人的探亲休假权利，推动建立子女护理假制度。建立完善社区老年人关爱服务机制。发展银发经济，推进智慧健康养老，满足老年妇女生活需要。依法保障老年妇女婚姻自由和家庭财产权利。
　　10．增强父母共同承担家庭教育责任的意识和能力。推进家庭教育立法及实施，促进父母共同落实家庭教育主体责任，创造有利于未成年子女健康成长和发展的家庭环境。开展宣传培训，帮助父母树立科学家庭教育理念，摒弃“重智轻德”等观念，掌握科学知识和方法，注重言传身教，关注未成年子女身心健康，提高家庭科学育儿能力。鼓励父母加强亲子交流，共同陪伴未成年子女成长。
　　（七）妇女与环境。
　　主要目标：
　　1．提高妇女的思想政治意识，引导妇女积极践行社会主义核心价值观。
　　2．提升全社会的性别平等意识，推进男女平等基本国策宣传教育进机关、进学校、进企业、进城乡社区、进家庭。
　　3．健全文化与传媒领域的性别平等评估和监管机制。
　　4．全面提升妇女的媒介素养，提高妇女利用信息技术参与新时代经济社会高质量发展的能力。
　　5．提高妇女的生态文明意识，促进妇女践行绿色发展理念，做生态文明建设的推动者和践行者。
　　6．减少环境污染对妇女健康的危害。农村自来水普及率达到90%，提升城市集中式饮用水水源水质。
　　7．稳步提高农村卫生厕所普及率，城镇公共厕所男女厕位比例标准化建设与实际需求相适应。
　　8．妇女应对突发事件能力不断提高，作用得到发挥，特殊需求得到满足。
　　9．广泛参与妇女领域的国际交流与合作，全面提升我国在国际妇女事务中的影响力。
　　策略措施：
　　1．加强对妇女的思想政治引领。坚持用习近平新时代中国特色社会主义思想引领妇女，持续开展中国特色社会主义和中国梦宣传教育，发挥新时代文明实践中心、主流媒体、妇女之家等阵地作用，推动理想信念教育常态化制度化，弘扬党和人民在各个历史时期奋斗中形成的伟大精神，激发妇女的历史责任感和主人翁精神，引导妇女听党话、跟党走，增强“四个意识”、坚定“四个自信”、做到“两个维护”。通过教育联系服务，凝聚青年女性、知识女性、新兴产业从业女性和活跃在网络空间中的女性。通过培养、评选、表彰、宣传妇女先进集体和个人，激励妇女崇尚先进、学习先进、争当先进。通过深化东中部地区与西部民族地区对口支援和交流合作，促进各族妇女广泛交往深度交融。
　　2．开展以男女平等为核心的先进性别文化宣传教育。将构建先进性别文化纳入繁荣社会主义先进文化制度体系。大力宣传新时代妇女在社会生活和家庭生活中的独特作用，宣传优秀妇女典型和性别平等优秀案例。推动各级干部学习习近平总书记关于妇女和妇女工作的重要论述以及马克思主义妇女观、男女平等基本国策。在机关、学校、企业、城乡社区、家庭以多种形式开展男女平等基本国策宣传教育，让性别平等成为全社会共同遵循的行为规范和价值标准。
　　3．促进妇女共建共享精神文明创建和城乡人居环境改善成果。丰富优质文化产品和公共文化服务供给，满足妇女精神文化需求。鼓励妇女积极参与城市文明建设，将妇女参与程度和满意度纳入文明城市评选内容。引导妇女在文明单位创建中爱岗敬业，争做文明职工。促进妇女参与文明村镇创建，主动参与农村人居环境整治提升、农村文化发展、文明乡风培育和乡村社会治理。推进城乡公共文化服务体系一体建设，创新实施文化惠民工程，惠及城乡妇女。
　　4．加强文化与传媒领域的性别平等培训、评估和监管。开展对文化传媒工作者和传媒相关专业学生的性别平等培训，提升文化与传媒领域性别平等传播能力。加强对公共文化产品和传媒涉及性别平等内容的监测和监管，吸纳性别专家参与相关评估，消除网络媒体、影视产品、公共出版物等中出现的歧视贬抑妇女、侮辱妇女人格尊严、物化妇女形象等不良现象，规范网络名人和公众账号传播行为。完善违规行为警示记录系统，优化线上舆情预警和线下评估处置机制。
　　5．引导妇女提高媒介素养。利用妇女之家、图书馆、网络课堂等开展面向妇女的媒介素养培训和指导，加强妇女网络素养教育，提升妇女对媒介信息选择、判断和有效利用的能力，提升妇女网络安全意识和能力，消除性别数字鸿沟。加强学生网络素养教育，引导女生合理安全使用网络，提升自我保护能力，防止网络沉迷。重点帮助老年妇女、困难妇女和残疾妇女群体掌握网络基本知识技能。开展争做“巾帼好网民”活动，推动妇女弘扬网上正能量。
　　6．充分发挥妇女在生态文明建设中的重要作用。广泛开展生态文化宣传教育和实践活动，引导妇女树立生态文明意识，提高环境科学素养，掌握环境科学知识，提升妇女生态环境保护意识和能力。鼓励妇女引领绿色生产生活，养成节约适度、绿色低碳、文明健康的生活方式和消费模式，杜绝浪费。支持妇女参与生态环境治理。
　　7．持续改善妇女生活的环境质量。加强生态环境监测和健康监测，开展环境污染因素影响研究，监测分析评估环境政策、基础设施项目、生产生活学习环境等对妇女健康的影响。推进城乡生活环境治理，推进城镇污水管网全覆盖，开发利用清洁能源，推行垃圾分类和减量化、资源化，推广使用节能环保产品。
　　8．为城乡妇女享有安全饮水提供保障。引导妇女积极参与水源保护。推进城市集中式饮用水水源地规范化建设，加强水源保护和水质监测，守护饮水安全命脉。加强水利基础设施建设，实施农村供水保障工程，提升水资源优化配置能力，为妇女取水、用水提供便利。
　　9．加强符合妇女需求的卫生厕所建设。推进城镇公共厕所改造，完善落实城镇公共厕所设计标准，推动将男女厕位比例规范化建设和达标率纳入文明城市、文明社区、文明村镇、文明单位、文明校园的评选标准。分类有序推进农村厕所革命，稳步提高卫生厕所普及率，加强厕所粪污无害化处理与资源化利用。推动旅游景区、商场、客运枢纽和服务区等公共场所建设第三卫生间。
　　10．在突发事件应对中关切妇女特别是孕期、哺乳期妇女及困难妇女群体的特殊需求。在突发事件应急体系建设、预防和应急处置机制建设、相关应急预案和规划制订中统筹考虑妇女特殊需求，优先保障女性卫生用品、孕产妇用品和重要医用物资供给。面向妇女开展突发事件预防应对知识和自救互救技能指导培训，提高妇女的防灾减灾意识和自救互救能力。在应对突发事件中加强对有需求妇女群体的救助服务和心理疏导。引导妇女积极参与防灾减灾工作。
　　11．积极促进国际妇女事务交流与合作。认真履行关于促进男女平等与妇女全面发展的国际公约和文件，积极落实联合国2030年可持续发展议程涉及性别平等相关目标。参与全球促进性别平等事业，提升我国的话语权和影响力，开展国际交流合作，促进妇女发展交流互鉴，讲好中国妇女发展故事，宣传中国妇女事业发展成就。积极主办和参与涉及妇女议题的各类国际会议，推动发展妇女民间外交，持续打造我国妇女人文交流品牌，在国际舞台上展现中国形象。支持妇女投身“一带一路”建设，为推动构建人类命运共同体发挥重要作用。
　　12．发挥妇联组织在营造男女平等和妇女全面发展环境中的积极作用。健全完善引领服务联系妇女的工作机制，发挥桥梁纽带作用，凝聚妇女人心。联合中央主流媒体，依托妇联全媒体，大力宣传习近平总书记关于妇女和妇女工作的重要论述，宣传马克思主义妇女观和男女平等基本国策，宣传妇女“半边天”作用。加强妇女舆情尤其是网络舆情监测，对错误观点言论及时发声，协调督促处置，正面引导舆论，优化有利于妇女全面发展的社会舆论环境。
　　（八）妇女与法律。
　　主要目标：
　　1．全面贯彻落实男女平等[宪法](https://www.pkulaw.com/chl/7c7e81f43957c58bbdfb.html)原则和基本国策，健全完善保障妇女合法权益的法律体系。
　　2．促进法规政策性别平等评估机制规范化建设和有效运行。
　　3．提高妇女尊法学法守法用法的意识和能力。充分发挥妇女在法治中国建设中的作用。
　　4．深入实施[反家庭暴力法](https://www.pkulaw.com/chl/ac57ba1df4413457bdfb.html)，预防和制止针对妇女一切形式的家庭暴力。
　　5．严厉打击拐卖妇女、性侵害妇女等违法犯罪行为。
　　6．提升预防和制止性骚扰的法治意识，有效遏制针对妇女的性骚扰。
　　7．严厉打击利用网络对妇女实施的违法犯罪行为。
　　8．保障妇女在家庭关系中的财产所有权、继承权，保障妇女对婚姻家庭关系中共同财产享有知情权和平等处理权。
　　9．依法为妇女提供公共法律服务。保障遭受侵害妇女获得及时有效的司法救助。
　　策略措施：
　　1．推进男女平等[宪法](https://www.pkulaw.com/chl/7c7e81f43957c58bbdfb.html)原则和基本国策贯彻落实到法治中国建设全过程。适时修订[妇女权益保障法](https://www.pkulaw.com/chl/f20e7cd055022d39bdfb.html)、[刑法](https://www.pkulaw.com/chl/39c1b78830b970eabdfb.html)、[社会保险法](https://www.pkulaw.com/chl/4449c7967540e761bdfb.html)、[女职工劳动保护特别规定](https://www.pkulaw.com/chl/cdf82292dbb62ee9bdfb.html)等法律法规，完善保障妇女合法权益的法律体系。加大[民法典](https://www.pkulaw.com/chl/aa00daaeb5a4fe4ebdfb.html)、[妇女权益保障法](https://www.pkulaw.com/chl/f20e7cd055022d39bdfb.html)等法律法规的实施力度，加强执法检查和督查督办，保障侵害妇女权益案件获得公平公正处理。促进开展妇女权益保障领域的公益诉讼。将保障妇女权益相关内容纳入基层社会治理，纳入法治队伍建设、全民普法规划和群众性法治文化活动，增强全社会的男女平等法治意识和法治素养。
　　2．加强法规政策性别平等评估工作。健全国家、省（自治区、直辖市）、市（地、州、盟）法规政策性别平等评估机制和县（市、区、旗）政策性别平等评估机制，明确评估范围，规范评估流程，细化评估指标。加强法规政策制定前研判、决策中贯彻、实施后评估的制度化建设。开展性别平等评估相关培训，加强专业化队伍建设，将男女平等基本国策落实到法规、规章、政策制定实施全过程各环节。
　　3．提升妇女法治意识和参与法治中国建设的能力。深入开展[民法典](https://www.pkulaw.com/chl/aa00daaeb5a4fe4ebdfb.html)等专项普法活动，面向妇女提供法律咨询等服务，引导妇女自觉学习[宪法](https://www.pkulaw.com/chl/7c7e81f43957c58bbdfb.html)和法律知识，增强法治观念，养成办事依法、遇事找法、解决问题用法、化解矛盾靠法的法治思维和行为习惯。鼓励妇女多途径参与立法、司法和普法活动。充分发挥女人大代表、女政协委员、妇联组织、以女性为成员主体或者以女性为主要服务对象的社会组织等在科学立法、民主立法和立法协商中的作用。
　　4．加大[反家庭暴力法](https://www.pkulaw.com/chl/ac57ba1df4413457bdfb.html)的实施力度。健全完善预防和制止家庭暴力多部门合作机制，适时出台落实[反家庭暴力法](https://www.pkulaw.com/chl/ac57ba1df4413457bdfb.html)的司法解释、指导意见或实施细则，发布反家庭暴力的典型案例或指导性案例。推动省（自治区、直辖市）、市（地、州、盟）出台反家庭暴力地方性法规。加强宣传教育、预防排查，建立社区网格化家庭暴力重点监控机制。完善落实家庭暴力发现、报告、处置机制，强化相关主体强制报告意识，履行强制报告义务。加大接处警工作力度，开展家庭暴力警情、出具告诫书情况统计。对构成犯罪的施暴人依法追究刑事责任，从严处理重大恶性案件。及时签发人身保护令，提高审核签发率，加大执行力度。加强紧急庇护场所管理，提升庇护服务水平。加强对家庭暴力受害妇女的心理抚慰和生活救助，帮助其身心康复。加强对施暴者的教育警示、心理辅导和行为矫治。开展家庭暴力案件跟踪回访。加强反家庭暴力业务培训和统计。
　　5．坚决打击拐卖妇女犯罪。完善落实集预防、打击、救助、安置、康复于一体的反拐工作长效机制。坚持预防为主、防治结合，提高全社会的反拐意识以及妇女的防范意识和能力。深入实施反对拐卖人口行动计划，打击拐卖妇女犯罪团伙。整治“买方市场”，及时解救被拐妇女并帮助其正常融入社会。打击跨国跨区域拐卖妇女犯罪。
　　6．加大对组织、强迫、引诱、容留、介绍卖淫等犯罪行为的打击力度。加强网络治理，利用大数据完善违法信息过滤、举报等功能，严厉打击利用网络组织、强迫、引诱、容留、介绍妇女卖淫。依法加大对强迫、引诱幼女和智力残疾妇女卖淫的打击力度。加强社会治安综合治理，建立常态化整治机制，鼓励群众监督和举报涉黄违法犯罪行为。
　　7．有效控制和严厉惩处强奸、猥亵、侮辱妇女特别是女童和智力、精神残疾妇女的违法犯罪行为。加强防性侵教育，提高妇女尤其是女童的防性侵意识和能力。建立完善重点人群和家庭关爱服务机制、侵权案件发现报告机制、多部门联防联动机制和侵权案件推进工作督查制度。完善立案侦查制度，及时、全面、一次性收集固定证据，避免受害妇女遭受“二次伤害”。建立性侵害违法犯罪人员信息查询系统，完善和落实从业禁止制度。加强对受害妇女的隐私保护、心理疏导和干预。
　　8．预防和制止针对妇女的性骚扰。推动完善防治性骚扰相关立法。多形式多渠道传播防治性骚扰知识，提升妇女防范和制止性骚扰的意识和能力。建立健全预防和制止性骚扰工作机制，加强联防联控，发挥典型案例示范指引作用。预防和制止公共场所和工作、学习等场所发生的性骚扰，在机关、企业、学校等单位建立相关工作机制，预防和制止利用职权、从属关系等实施性骚扰。畅通救济途径。
　　9．保障妇女免遭利用网络实施违法犯罪行为的侵害。加强网络信息内容生态治理，加强对网络淫秽色情信息的监管和查处，依法打击网络信息服务平台、生产者和使用者对妇女实施猥亵、侮辱、诽谤、性骚扰、散布谣言、侵犯隐私等违法犯罪行为。加强对网络平台的规范管理，保护妇女个人信息安全。依法惩治利用网络非法收集、使用、加工、传输、买卖、提供或者公开妇女个人信息的违法犯罪行为。提高妇女防范电信网络诈骗的意识和能力，严厉打击采取非法网络贷款、虚假投资、咨询服务等手段骗取妇女钱财的违法犯罪行为。
　　10．在婚姻家庭和继承案件处理中依法保障妇女的财产权益。保障妇女平等享有家庭财产的占有、使用、收益和处分权利。保障妇女依法享有夫妻互相继承遗产、子女平等继承遗产的权利。保障夫妻对共同财产享有平等的知情权、处理权，认定和分割夫妻共同财产、认定和清偿夫妻共同债务时，切实保障妇女合法权益。离婚时，保障妇女依法获得土地、房屋、股份等权益，保障负担较多家庭义务的妇女获得补偿、生活困难妇女获得经济帮助、无过错妇女依法获得损害赔偿。
　　11．为妇女提供优质高效的公共法律服务。推进公共法律服务实体、网络、热线三大平台融合发展，为妇女特别是低收入妇女、老年妇女、残疾妇女、单亲困难母亲等提供便捷高效、均等普惠的公共法律服务。落实法律法规对妇女申请法律援助的相关规定，保障妇女在刑事、民事、行政案件中享有诉讼代理和维权指导服务。加强维护妇女合法权益的法律援助类社会组织和专业律师、基层法务工作者队伍建设。保障特定案件中生活困难妇女能够获得司法救助。
　　12．发挥妇联组织代表和维护妇女合法权益的职能作用。支持妇联组织健全联合约谈、联席会议、信息通报、调研督查、发布案例等工作制度，推动保障妇女权益法律政策的制定实施。加强“12338”妇女维权热线建设，畅通妇女有序表达诉求的渠道。及时发现报告侵权问题，依法建议查处性别歧视事件或协助办理侵害妇女权益案件，配合打击侵害妇女合法权益的违法犯罪行为，为受侵害妇女提供帮助。

　　**三、**组织实施
　　（一）坚持党的全面领导。坚持以习近平新时代中国特色社会主义思想为指导，坚持以人民为中心的发展思想，坚持走中国特色社会主义妇女发展道路，把党的领导贯穿于纲要组织实施全过程。贯彻党中央关于妇女事业发展的决策部署，坚持和完善促进男女平等和妇女全面发展的制度机制，在统筹推进“五位一体”总体布局、协调推进“四个全面”战略布局中推进纲要实施。
　　（二）落实纲要实施责任。完善落实党委领导、政府主责、妇儿工委协调、多部门合作、全社会参与的纲要实施工作机制。国务院及地方各级人民政府负责纲要实施工作，各级妇儿工委负责组织、协调、指导、督促工作，各级妇儿工委办公室负责具体工作。有关部门、相关机构和人民团体结合职责，承担纲要相关目标任务落实工作。在出台法律、制定政策、编制规划、部署工作时贯彻落实男女平等基本国策，切实保障妇女合法权益，促进妇女全面发展。
　　（三）加强纲要与国民经济和社会发展规划的衔接。在经济社会发展总体规划及相关专项规划中贯彻落实男女平等基本国策，将纲要实施以及妇女事业发展纳入经济社会发展总体规划及相关专项规划，结合经济社会发展总体规划部署要求推进纲要实施，实现妇女事业发展与经济社会发展同步规划、同步部署、同步推进、同步落实。
　　（四）制定地方妇女发展规划和部门实施方案。省级人民政府依据本纲要，结合实际制定本级妇女发展规划。市、县级人民政府依据本纲要以及上一级妇女发展规划，结合实际制定本级妇女发展规划。省、市、县级规划颁布后1个月内报送上一级妇儿工委办公室。中央及地方承担纲要（规划）目标任务的有关部门、相关机构和人民团体结合职责，按照任务分工，制定实施方案并报送同级妇儿工委办公室。
　　（五）完善实施纲要的工作制度机制。健全目标管理责任制，将纲要实施纳入政府议事日程和考核内容，将纲要目标分解到责任单位并纳入目标管理和考核内容。健全督导检查制度，定期对纲要实施情况开展督查。健全报告制度，责任单位每年向同级妇儿工委报告纲要实施情况和下一年工作安排，下级妇儿工委每年向上一级妇儿工委报告本地区规划实施情况和下一年工作安排。健全议事协调制度，定期召开妇女儿童工作会议和妇儿工委全体会议、联络员会议等，总结交流情况，研究解决问题，部署工作任务。健全纲要实施示范制度，充分发挥示范单位以点带面、示范带动作用。健全表彰制度，对实施纲要先进集体和先进个人按照有关规定进行表彰。
　　（六）加强妇女发展经费支持。各级人民政府将实施纲要所需工作经费纳入财政预算，实现妇女事业和经济社会同步发展。重点支持革命老区、民族地区、边疆地区、欠发达地区妇女发展，支持特殊困难妇女群体发展。动员社会力量，多渠道筹集资源，共同发展妇女事业。
　　（七）坚持和创新实施纲要的有效做法。贯彻新发展理念，坚持问题导向、目标导向，构建促进妇女发展的法律法规政策体系，完善妇女合法权益保障机制，实施促进妇女发展的民生项目。通过分类指导、示范先行，总结推广好做法好经验。通过政府购买服务等方式，发挥社会力量推动纲要实施的作用。开展国际交流合作，交流互鉴经验做法，讲好中国妇女发展故事。
　　（八）加强纲要实施能力建设。将习近平总书记关于妇女和妇女工作的重要论述以及男女平等基本国策有关内容、相关法律法规政策纳入各级干部学习内容，将实施纲要所需知识纳入培训计划，举办多层次、多形式培训，增强政府有关部门、相关机构和人员实施纲要的责任意识和能力。以政治建设为统领，加强各级妇儿工委及其办公室能力建设，促进机构职能优化高效，为更好履职尽责提供必要的人力物力财力支持，为纲要实施提供组织保障。
　　（九）加大纲要宣传力度。大力宣传习近平总书记关于妇女和妇女工作的重要论述，宣传在党的坚强领导下妇女事业发展的成就，宣传男女平等基本国策和保障妇女合法权益、促进妇女发展的法律法规政策，宣传纲要内容和纲要实施的经验、成效，努力营造有利于妇女发展的社会氛围。
　　（十）加强妇女发展调查研究。充分发挥各级妇儿工委及其办公室作用，加强妇女发展专家队伍建设，依托高校、研究机构、社会组织等建设妇女发展研究基地，培育专业研究力量，广泛深入开展理论与实践研究，为制定完善相关法律法规政策提供参考。
　　（十一）鼓励社会各界广泛参与纲要实施。鼓励企事业单位、社会组织、慈善机构和公益人士参与保障妇女合法权益、促进妇女发展等工作。鼓励妇女参与纲要实施，提高妇女在参与纲要实施中实现自身全面发展的意识和能力。

　　**四、**监测评估
　　（一）加强监测评估制度建设。对纲要实施情况进行年度监测、中期评估、终期评估。落实并逐步完善性别统计监测方案。各级统计部门牵头组织开展年度监测，各级妇儿工委成员单位、有关部门、相关机构向同级统计部门报送年度监测数据，及时收集、分析反映妇女发展状况的相关数据和信息。各级妇儿工委组织开展中期和终期评估，各级妇儿工委成员单位、有关部门、相关机构向同级妇儿工委提交中期和终期评估报告。通过评估，了解掌握纲要实施进展和妇女发展状况，系统分析评价纲要目标任务完成情况，评判纲要策略措施的实施效果，总结经验做法，找出突出问题，预测发展趋势，提出对策建议。监测评估工作所需经费纳入财政预算。
　　（二）加强监测评估工作组织领导。各级妇儿工委设立监测评估领导小组，由同级妇儿工委及相关部门负责同志组成，负责监测评估工作的组织领导、监测评估方案的审批、监测评估报告的审核等。领导小组下设监测组和评估组。
　　监测组由各级统计部门牵头，相关部门负责纲要实施情况统计监测的人员参加，负责监测工作的组织、指导和培训，制定监测方案和指标体系，收集、分析数据信息，向同级妇儿工委提交年度、中期和终期监测报告，编辑出版年度妇女儿童统计资料等。监测组成员负责统筹协调本部门纲要实施监测、分析、数据上报、分性别分年龄指标完善等工作。
　　评估组由各级妇儿工委办公室牵头，相关部门负责纲要实施的人员参加，负责评估工作的组织、指导和培训，制定评估方案，组织开展评估工作，向同级妇儿工委提交中期和终期评估报告。评估组成员负责统筹协调本部门纲要实施自我评估工作，参加妇儿工委组织的评估工作。支持评估组相关部门就妇女保护与发展中的突出问题开展专项调查、评估，结果可供中期和终期评估参考。
　　（三）加强分性别统计监测。规范完善性别统计监测指标体系，根据需要调整扩充妇女发展统计指标，推动纳入国家和部门常规统计以及统计调查制度，加强部门分性别统计工作，推进分性别统计监测制度化建设。国家、省、市三级建立完善妇女发展统计监测数据库，支持县级妇女发展统计监测数据库建设。鼓励支持相关部门对妇女发展缺项数据开展专项统计调查。
　　（四）提升监测评估工作能力和水平。加强监测评估工作培训和部门协作，规范监测数据收集渠道、报送方式，提高数据质量。运用互联网和大数据等，丰富分性别统计信息。科学设计监测评估方案和方法，探索开展第三方评估。提升监测评估工作科学化、标准化、专业化水平。
　　（五）有效利用监测评估成果。发挥监测评估结果服务决策的作用，定期向同级人民政府及相关部门报送监测评估情况，为决策提供依据。建立监测评估报告交流、反馈和发布机制。加强对监测评估结果的研判和运用，对预计完成困难、波动较大的监测指标及时预警，对评估发现的突出问题和薄弱环节及时提出对策建议。运用监测评估结果指导下一阶段纲要实施，实现纲要实施的常态化监测、动态化预警、精准化干预、高质量推进。

中国儿童发展纲要（2021-2030年）
前　　言

　　儿童是国家的未来、民族的希望。当代中国少年儿童既是实现第一个百年奋斗目标的经历者、见证者，更是实现第二个百年奋斗目标、建设社会主义现代化强国的生力军。促进儿童健康成长，能够为国家可持续发展提供宝贵资源和不竭动力，是建设社会主义现代化强国、实现中华民族伟大复兴中国梦的必然要求。党和国家始终高度重视儿童事业发展，先后制定实施三个周期的中国儿童发展纲要，为儿童生存、发展、受保护和参与权利的实现提供了重要保障。
　　党的十八大以来，以习近平同志为核心的党中央把培养好少年儿童作为一项战略性、基础性工作，坚持儿童优先原则，大力发展儿童事业，保障儿童权利的法律法规政策体系进一步完善，党委领导、政府主责、妇女儿童工作委员会（以下简称妇儿工委）协调、多部门合作、全社会参与的儿童工作机制进一步巩固，儿童发展环境进一步优化。截至2020年底，婴儿、5岁以下儿童死亡率分别从2010年的13.1‰、16.4‰下降到5.4‰、7.5‰；学前教育毛入园率从2010年的56.6%上升到85.2%，九年义务教育巩固率从2010年的91.1%上升到95.2%，高中阶段毛入学率从2010年的82.5%上升到91.2%；农村留守儿童、困境儿童等弱势群体得到更多关爱和保护。儿童发展和儿童事业取得了历史性新成就。
　　受经济社会发展水平制约，我国儿童事业发展仍然存在不平衡不充分问题。贯彻儿童优先原则的力度需要进一步加大，儿童思想引领需要进一步增强，保障儿童权利的法治建设需要持续推进，儿童发展的城乡、区域和群体之间差距需要进一步缩小，基层儿童保护和服务机制需要进一步健全，科技进步和生活方式变革给做好儿童工作带来新挑战，儿童事业发展使命艰巨、任重道远。
　　当前，我国正处于实现“两个一百年”奋斗目标的历史交汇期。坚持党的全面领导，坚持以人民为中心，坚持新发展理念，统筹推进“五位一体”总体布局，协调推进“四个全面”战略布局，推进国家治理体系和治理能力现代化，构建人类命运共同体，为儿童事业发展提供了重大机遇、擘画了美好前景。站在新的历史起点上，需要进一步落实儿童优先原则，全面提高儿童综合素质，培养造就德智体美劳全面发展的社会主义建设者和接班人，引领亿万儿童勇担新使命、建功新时代。
　　依据[宪法](https://www.pkulaw.com/chl/7c7e81f43957c58bbdfb.html)和[未成年人保护法](https://www.pkulaw.com/chl/883cecd7fe38636dbdfb.html)等有关法律法规，按照国家经济社会发展的总体目标和要求，结合我国儿童发展的实际情况，参照联合国《儿童权利公约》和2030年可持续发展议程等国际公约和文件宗旨，制定本纲要。

　　**一、**指导思想、基本原则和总体目标
　　（一）指导思想。
　　高举中国特色社会主义伟大旗帜，深入贯彻党的十九大和十九届二中、三中、四中、五中全会精神，坚持以马克思列宁主义、毛泽东思想、邓小平理论、“三个代表”重要思想、科学发展观、习近平新时代中国特色社会主义思想为指导，坚定不移贯彻新发展理念，坚持以人民为中心的发展思想，坚持走中国特色社会主义儿童发展道路，坚持和完善最有利于儿童、促进儿童全面发展的制度机制，落实立德树人根本任务，优化儿童发展环境，保障儿童生存、发展、受保护和参与权利，全面提升儿童综合素质，为实现第二个百年奋斗目标、建设社会主义现代化强国奠定坚实的人才基础。
　　（二）基本原则。
　　1．坚持党的全面领导。把握儿童事业发展的政治方向，贯彻落实党中央关于儿童事业发展的决策部署，切实把党的领导贯彻到儿童事业发展的全过程和各方面。
　　2．坚持对儿童发展的优先保障。在出台法律、制定政策、编制规划、部署工作时优先考虑儿童的利益和发展需求。
　　3．坚持促进儿童全面发展。尊重儿童的人格尊严，遵循儿童身心发展特点和规律，保障儿童身心健康，促进儿童在德智体美劳各方面全面发展。
　　4．坚持保障儿童平等发展。创造公平社会环境，消除对儿童一切形式的歧视，保障所有儿童平等享有发展权利和机会。
　　5．坚持鼓励儿童参与。尊重儿童主体地位，鼓励和支持儿童参与家庭、社会和文化生活，创造有利于儿童参与的社会环境。
　　（三）总体目标。
　　保障儿童权利的法律法规政策体系更加健全，促进儿童发展的工作机制更加完善，儿童优先的社会风尚普遍形成，城乡、区域、群体之间的儿童发展差距明显缩小。儿童享有更加均等和可及的基本公共服务，享有更加普惠和优越的福利保障，享有更加和谐友好的家庭和社会环境。儿童在健康、安全、教育、福利、家庭、环境、法律保护等领域的权利进一步实现，思想道德素养和全面发展水平显著提升，获得感、幸福感、安全感明显增强。展望2035年，与国家基本实现社会主义现代化相适应，儿童优先原则全面贯彻，儿童全面发展取得更为明显的实质性进展，广大儿童成长为建设社会主义现代化强国、担当民族复兴大任的时代新人。

　　**二、**发展领域、主要目标和策略措施
　　（一）儿童与健康。
　　主要目标：
　　1．覆盖城乡的儿童健康服务体系更加完善，儿童医疗保健服务能力明显增强，儿童健康水平不断提高。
　　2．普及儿童健康生活方式，提高儿童及其照护人健康素养。
　　3．新生儿、婴儿和5岁以下儿童死亡率分别降至3.0‰、5.0‰和6.0‰以下，地区和城乡差距逐步缩小。
　　4．构建完善覆盖婚前、孕前、孕期、新生儿和儿童各阶段的出生缺陷防治体系，预防和控制出生缺陷。
　　5．儿童常见疾病和恶性肿瘤等严重危害儿童健康的疾病得到有效防治。
　　6．适龄儿童免疫规划疫苗接种率以乡（镇、街道）为单位保持在90%以上。
　　7．促进城乡儿童早期发展服务供给，普及儿童早期发展的知识、方法和技能。
　　8．5岁以下儿童贫血率和生长迟缓率分别控制在10%和5%以下，儿童超重、肥胖上升趋势得到有效控制。
　　9．儿童新发近视率明显下降，小学生近视率降至38%以下，初中生近视率降至60%以下，高中阶段学生近视率降至70%以下。0-6岁儿童眼保健和视力检查覆盖率达到90%以上。
　　10．增强儿童体质，中小学生国家学生体质健康标准达标优良率达到60%以上。
　　11．增强儿童心理健康服务能力，提升儿童心理健康水平。
　　12．适龄儿童普遍接受性教育，儿童性健康服务可及性明显提高。
　　策略措施：
　　1．优先保障儿童健康。将儿童健康理念融入经济社会发展政策，儿童健康主要指标纳入政府目标和责任考核。完善涵盖儿童的基本医疗卫生制度，加强儿童医疗保障政策与公共卫生政策衔接。加大对儿童医疗卫生与健康事业的投入力度，支持革命老区、民族地区、边疆地区和欠发达地区的儿童健康事业发展，逐步实现基本妇幼健康服务均等化。建设统一的妇幼健康信息平台，推动妇幼健康信息平台与电子健康档案的互联互通和信息共享，完善妇幼健康统计调查制度，推行“互联网＋妇幼健康”服务模式，完善妇幼健康大数据，加强信息互联共享，实现儿童健康全周期全过程管理和服务的信息化、智能化。开展“儿童健康综合发展示范县”创建活动。
　　2．完善儿童健康服务体系。构建国家、区域、省、市、县级儿童医疗保健服务网络，以妇幼保健机构、儿童医院和综合医院儿科为重点，统筹规划和配置区域内儿童健康服务资源。省、市、县级均各设置1所政府举办、标准化的妇幼保健机构，每千名儿童拥有儿科执业（助理）医生达到1.12名、床位增至3.17张。建立完善以区县妇幼保健机构为龙头，乡镇卫生院、社区卫生服务中心为枢纽，村卫生室为基础的基层儿童保健服务网络，每所乡镇卫生院、社区卫生服务中心至少配备1名提供规范儿童基本医疗服务的全科医生，至少配备2名专业从事儿童保健的医生。完善儿童急救体系。加快儿童医学人才培养，提高全科医生的儿科和儿童保健专业技能，提高儿科医务人员薪酬待遇。
　　3．加大儿童健康知识宣传普及力度。强化父母或其他监护人是儿童健康第一责任人的理念，依托家庭、社区、学校、幼儿园、托育机构，加大科学育儿、预防疾病、及时就医、合理用药、合理膳食、应急避险、心理健康等知识和技能宣传普及力度，促进儿童养成健康行为习惯。构建全媒体健康知识传播机制。发挥健康科普专家库和资源库作用。推进医疗机构规范设置“孕妇学校”和家长课堂，鼓励医疗机构、医务人员、相关社会组织等开展健康科普活动。预防和制止儿童吸烟（含电子烟）、酗酒，保护儿童远离毒品。
　　4．保障新生儿安全与健康。深入实施危重新生儿筛查与评估、高危新生儿专案管理、危急重症救治、新生儿死亡评审等制度。加强新生儿规范化访视工作，新生儿访视率保持在90%以上。完善医疗机构产科、新生儿科质量规范化管理体系，加强新生儿保健专科建设。依托现有机构加强危重新生儿救治中心建设，强化危重新生儿救治保障。
　　5．加强出生缺陷综合防治。建立多部门联动防治出生缺陷的工作机制，落实出生缺陷三级防治措施，加强知识普及和出生缺陷防控咨询，推广婚姻登记、婚育健康宣传教育、生育指导“一站式”服务。强化婚前孕前保健，提升产前筛查和诊断能力，推动围孕期、产前产后一体化和多学科诊疗协作，规范服务与质量监管。扩大新生儿疾病筛查病种范围，建立筛查、阳性病例召回、诊断、治疗和随访一体化服务模式，促进早筛早诊早治。加强地中海贫血防治。健全出生缺陷防治网络，加强出生缺陷监测，促进出生缺陷防治领域科技创新和成果转化。
　　6．加强儿童保健服务和管理。加强儿童保健门诊标准化、规范化建设，提升儿童保健服务质量。扎实开展0-6岁儿童健康管理工作，3岁以下儿童系统管理率和7岁以下儿童健康管理率保持在90%以上。推进以视力、听力、肢体、智力及孤独症等五类残疾为重点的0-6岁儿童残疾筛查，完善筛查、诊断、康复、救助相衔接的工作机制。提高儿童康复服务能力和水平。增强学校、幼儿园、托育机构的常见病预防保健能力，按标准配备校医、幼儿园及托育机构卫生保健人员和必要保健设备。加强对孤儿、流动儿童、留守儿童和困境儿童等重点人群的健康管理。
　　7．强化儿童疾病防治。以早产、低出生体重、贫血、肥胖、心理行为异常、视力不良、龋齿等儿童健康问题为重点，推广儿童疾病防治适宜技术，建立早期筛查、诊断和干预服务机制。加强儿童口腔保健，12岁儿童龋患率控制在25%以内。加强儿童重大传染性疾病、新发传染病管理以及艾滋病、梅毒、乙肝母婴阻断工作。完善儿童血液病、恶性肿瘤等重病诊疗体系、药品供应制度、综合保障制度，开发治疗恶性肿瘤等疾病的特效药。科学合理制定罕见病目录，加强罕见病管理。推广应用中医儿科适宜技术。
　　8．加强儿童免疫规划疫苗管理和预防接种。扩大国家免疫规划，维持较高水平的国家免疫规划疫苗接种率。支持多联多价等新型疫苗研制。加强疫苗研制、生产、流通和预防接种管理。完善预防接种异常反应补偿相关政策。
　　9．加强儿童早期发展服务。建立健全多部门协作的儿童早期发展工作机制，开展涵盖良好健康、充足营养、回应性照护、早期学习、安全保障等多维度的儿童早期发展综合服务。加强对家庭和托育机构的婴幼儿早期发展指导服务。促进儿童早期发展服务进农村、进社区、进家庭，探索推广入户家访指导等适合农村边远地区儿童、困境儿童的早期发展服务模式。
　　10．改善儿童营养状况。关注儿童生命早期1000天营养，开展孕前、孕产期营养与膳食评价指导。实施母乳喂养促进行动，强化爱婴医院管理，加强公共场所和工作场所母婴设施建设，6个月内婴儿纯母乳喂养率达到50%以上。普及为6月龄以上儿童合理添加辅食的知识技能。开展儿童生长发育监测和评价，加强个性化营养指导，保障儿童营养充足。加强食育教育，引导科学均衡饮食、吃动平衡，预防控制儿童超重和肥胖。加强学校、幼儿园、托育机构的营养健康教育和膳食指导。加大碘缺乏病防治知识宣传普及力度。完善食品标签体系。
　　11．有效控制儿童近视。加强0-6岁儿童眼保健和视力检查工作，推动建立儿童视力电子档案。减轻学生学业负担，指导监督学生做好眼保健操，纠正不良读写姿势。保障学校、幼儿园、托育机构室内采光、照明和课桌椅、黑板等达到规定标准。指导家长掌握科学用眼护眼知识并引导儿童科学用眼护眼。教育儿童按需科学规范合理使用电子产品。确保儿童每天接触户外自然光不少于1小时。
　　12．增强儿童身体素质。推进阳光体育运动，开足开齐体育与健康课。保障儿童每天至少1小时中等及以上强度的运动，培养儿童良好运动习惯。全面实施《国家学生体质健康标准》，完善学生健康体检和体质监测制度。鼓励公共体育场馆设施免费或优惠向周边学校和儿童开放，落实学校体育场馆设施在课余和节假日向学生开放政策，支持学校向体育类社会组织购买课后体育服务。进一步加大户外运动、健身休闲等配套公共基础设施建设力度。合理安排儿童作息，保证每天睡眠时间小学生达到10小时、初中生达到9小时、高中生达到8小时。
　　13．加强儿童心理健康服务。构建儿童心理健康教育、咨询服务、评估治疗、危机干预和心理援助公共服务网络。中小学校配备心理健康教育教师。积极开展生命教育和挫折教育，培养儿童珍爱生命意识和自我情绪调适能力。关注和满足孤儿、事实无人抚养儿童、留守儿童和困境儿童心理发展需要。提高教师、家长预防和识别儿童心理行为异常的能力，加强儿童医院、精神专科医院和妇幼保健机构儿童心理咨询及专科门诊建设。大力培养儿童心理健康服务人才。
　　14．为儿童提供性教育和性健康服务。引导儿童树立正确的性别观念和道德观念，正确认识两性关系。将性教育纳入基础教育体系和质量监测体系，增强教育效果。引导父母或其他监护人根据儿童年龄阶段和发展特点开展性教育，加强防范性侵害教育，提高儿童自我保护意识和能力。促进学校与医疗机构密切协作，提供适宜儿童的性健康服务，保护就诊儿童隐私。设立儿童性健康保护热线。
　　15．加强儿童健康领域科研创新。围绕儿童重大疾病的预防、诊断、治疗、康复和健康管理开展基础研究和应用研究。加强儿科科技创新基地、平台建设，发挥儿科医学领域国家临床医学研究中心重要作用。鼓励儿童用药研发生产，加快儿童用药申报审批工作。完善儿童临床用药规范，药品说明书明确表述儿童用药信息。扩大[国家基本药物目录](https://www.pkulaw.com/chl/cb8df04c7cbc07d4bdfb.html)中儿科用药品种和剂型范围，探索制定国家儿童基本药物目录，及时更新儿童禁用药品目录。推动儿童健康科技国际交流合作。
　　（二）儿童与安全。
　　主要目标：
　　1．减少儿童伤害所致死亡和残疾。儿童伤害死亡率以2020年数据为基数下降20%。
　　2．排查消除溺水隐患，儿童溺水死亡率持续下降。
　　3．推广使用儿童安全座椅、安全头盔，儿童出行安全得到有效保障。
　　4．减少儿童跌倒、跌落、烧烫伤和中毒等伤害的发生、致残和死亡。
　　5．儿童食品安全得到有效保障。
　　6．提升儿童用品质量安全水平。
　　7．预防和制止针对儿童一切形式的暴力。
　　8．提高对学生欺凌的综合治理能力，预防和有效处置学生欺凌。
　　9．预防和干预儿童沉迷网络，有效治理不良信息、泄露隐私等问题。
　　10．儿童遭受意外和暴力伤害的监测报告系统进一步完善。
　　策略措施：
　　1．创建儿童安全环境。树立儿童伤害可防可控意识，通过宣传教育、改善环境、加强执法、使用安全产品、开展评估等策略，创建有利于儿童成长的家庭、学校、社区安全环境。开展安全自护教育，提高儿童及其看护人的安全意识，帮助其掌握安全知识技能，培养儿童安全行为习惯。落实学校、幼儿园、托育机构等安全管理主体责任，开展儿童防伤害、防暴力、避灾险、会自救等教育活动。
　　2．建立健全儿童伤害防控工作体系。加大儿童伤害防控领域立法和执法力度。构建完善多部门合作的儿童伤害防控工作机制，鼓励社会力量参与儿童伤害防控。制定实施国家、地方儿童伤害防控行动计划，探索创新并大力推广儿童伤害防控适宜技术，优先制定实施针对流动儿童、留守儿童、困境儿童的伤害防控措施。
　　3．预防和控制儿童溺水。加强看护，保证儿童远离危险水体。隔离、消除家庭及校园环境的溺水隐患，加强农村地区相关水体的防护隔离和安全巡查，加强开放性水域、水上游乐场所、船只等安全管理并配置适用于儿童的应急救援装备。加强预防溺水和应急救援知识技能教育，普及儿童游泳及水上安全技能，引导儿童使用安全游泳场所。
　　4．预防和控制儿童道路交通伤害。完善交通安全立法。提高儿童看护人看护能力，培养儿童养成良好交通行为习惯。推广使用儿童安全座椅、安全头盔和儿童步行及骑乘非机动车反光标识。完善儿童道路安全防护用品标准，加强生产和销售监管。道路规划建设充分考虑儿童年龄特点，完善校园周边安全设施，严查严处交通违法行为。落实[校车安全管理条例](https://www.pkulaw.com/chl/79525d653e982af9bdfb.html)。
　　5．预防和控制儿童跌倒、跌落、烧烫伤、中毒等伤害。消除环境危险因素，推广使用窗户护栏、窗口限位器等防护用品，减少儿童跌倒、跌落。教育儿童远离火源，引导家庭分隔热源，安全使用家用电器，推广使用具有儿童保护功能的家用电器，预防儿童烧烫伤。推广使用儿童安全包装，提升儿童看护人对农药、药物、日用化学品等的识别及保管能力，避免儿童中毒。预防婴幼儿窒息，提升看护人对婴幼儿有效照护能力。规范犬类管理及宠物饲养，预防动物咬伤。加强防灾减灾教育，提高儿童及其看护人针对地震、火灾、踩踏等的防灾避险技能。
　　6．加强儿童食品安全监管。完善儿童食品安全标准体系。强化婴幼儿配方食品和婴幼儿辅助食品安全监管，加大婴幼儿配方乳粉产品抽检监测及不合格食品处罚力度。落实学校、幼儿园、托育机构食品安全管理主体责任，消除儿童集体用餐各环节食品安全隐患，加强校内及周边食品安全监管。严肃查处食品安全违法违规行为。
　　7．预防和减少产品引发的儿童伤害。强化产品质量安全监管。健全儿童用品强制性国家标准体系，完善产品安全警示标识。建立健全儿童玩具强制性国家标准，加强儿童用品行业自律，鼓励制定技术先进的团体标准。对部分儿童用品依法实施强制性产品认证管理。持续开展儿童用品质量安全守护行动，严厉查处制售假冒伪劣产品的违法行为，鼓励消费者依法投诉举报产品安全问题。加强对产品造成儿童伤害的信息监测、分析、监督检查和缺陷产品召回工作。杜绝“毒跑道”、“毒校服”，保障游戏游艺设备及大型游乐设施安全，引导儿童安全使用电动扶梯、旋转门等设施设备。
　　8．预防和控制针对儿童的暴力伤害。宣传倡导对儿童暴力零容忍理念，提升公众的法治意识和儿童保护意识，增强儿童的安全意识和自我保护能力。强化国家、社会、学校、家庭保护责任，建立防控针对儿童暴力伤害的部门合作工作机制，健全各级未成年人保护工作平台，落实针对儿童暴力伤害的发现、报告、干预机制。落实密切接触未成年人的机构和人员的强制报告责任。鼓励公众依法劝阻、制止、检举、控告针对儿童的暴力行为。依法严惩针对儿童的暴力违法犯罪行为。
　　9．加强对学生欺凌的综合治理。完善落实学生欺凌综合治理的部门合作工作机制。营造文明安全校园环境，加强思想道德教育、法治教育和心理健康教育，培养学生的健全人格和社会交往能力。严格学校日常安全管理，健全学生欺凌早期预警、事中处理、事后干预等工作机制，提高教职员工、家长、学生对欺凌的预防和处置能力。依法依规调查和处置欺凌事件，发挥教育惩戒作用。强化校园周边综合治理，将学生欺凌专项治理纳入社会治安综合治理工作。
　　10．加强未成年人网络保护。落实政府、企业、学校、家庭、社会保护责任，为儿童提供安全、健康的网络环境，保障儿童在网络空间中的合法权益。加强网络监管和治理，完善和落实网络信息监测、识别、举报、处置制度，依法惩处利用网络散布价值导向不良信息、从事危害未成年人身心健康的行为。网络游戏、网络直播、网络音视频、网络社交等网络服务提供者，针对未成年人使用其服务依法设置相应的时间管理、权限管理、消费管理等功能，不得为未满十六周岁儿童提供网络直播发布者账号注册服务。加强网络语言文明教育，坚决遏阻庸俗暴戾网络语言传播。实施国家统一的未成年人网络游戏电子身份认证，完善游戏产品分类、内容审核、时长限制等措施。加强儿童个人信息和隐私保护。
　　11．提高对儿童遭受意外和暴力伤害的紧急救援、医疗救治、康复服务水平。广泛宣传儿童紧急救援知识，提升看护人、教师紧急救援技能。完善公共场所急救设施配备。完善国家紧急医学救援网络，加强儿童伤害院前急救设施设备配备，实现院前急救与院内急诊的有效衔接，加强康复机构能力建设，提高儿童医学救治以及康复服务的效率和水平。
　　12．完善监测机制。建立健全国家和地方儿童遭受意外和暴力伤害监测体系，通过医疗机构、学校、幼儿园、托育机构、社区、司法机关等多渠道收集儿童伤害数据，促进数据规范化。建立多部门、多专业参与的数据共享、分析、评估和利用工作机制。
　　（三）儿童与教育。
　　主要目标：
　　1．全面落实立德树人根本任务，培养德智体美劳全面发展的社会主义建设者和接班人。
　　2．适龄儿童普遍接受有质量的学前教育，学前教育毛入园率达到并保持在90%以上。
　　3．促进义务教育优质均衡发展和城乡一体化，九年义务教育巩固率提高到96%以上。
　　4．巩固提高高中阶段教育普及水平，高中阶段教育毛入学率达到并保持在92%以上。
　　5．孤儿、事实无人抚养儿童、残疾儿童、农业转移人口随迁子女、留守儿童、困境儿童等特殊群体受教育权利得到根本保障。残疾儿童义务教育巩固水平进一步提高。
　　6．儿童科学素质全面提升，科学兴趣、创新意识、实践能力不断提高。
　　7．以提高儿童综合素质为导向的教育评价体系更加完善。
　　8．加强校园文化建设，营造友善、平等、相互尊重的师生关系和同学关系。
　　9．学校家庭社会协同育人机制进一步完善。
　　策略措施：
　　1．全面贯彻党的教育方针。坚持社会主义办学方向，健全立德树人落实机制，实施素质教育，完善德智体美劳全面培养的教育体系。提高思想道德素质，引领学生坚定理想信念，热爱祖国、热爱人民、热爱中国共产党、热爱社会主义，培育和践行社会主义核心价值观。提升智育水平，发展学生终身学习能力，促进思维发展，激发创新意识。坚持健康第一，深化体教融合，帮助学生磨练坚强意志、锻炼强健体魄。改进美育教学，提升学生审美和人文素养。加强劳动教育，引导学生树立正确的劳动观，形成良好劳动习惯，培养勤俭、奋斗、创新、奉献的劳动精神。
　　2．全面落实教育优先发展战略。经济社会发展规划上优先安排教育，财政资金投入上优先保障教育，公共资源配置上优先满足教育。优化教育经费支出结构，把义务教育作为教育投入重中之重。依法落实各级政府教育支出责任，完善各教育阶段财政补助政策。支持和规范社会力量办学。
　　3．培养儿童良好思想道德素质、法治意识和行为习惯。加强理想教育、道德教育、法治教育、劳动教育，养成良好道德品质、法治意识和行为习惯，形成积极健康的人格和良好心理品质。中小学、幼儿园广泛开展性别平等教育。完善德育工作体系，深化课程育人、文化育人、活动育人、实践育人、管理育人、协同育人。创新德育工作形式，丰富德育内容，增强德育工作吸引力、感染力和实效性。
　　4．全面推进教育理念、体系、制度、内容、方法创新。严格落实课程方案和课程标准，提高教学质量。完善教材编写、修订、审查、选用、退出机制。遵循教育规律和学生身心发展规律，尊重个体差异，因材施教，推行启发式、探究式、参与式、合作式教学。探索具有特殊才能学生的培养体系。提升校园智能化水平，提高信息化服务教育教学和管理的能力水平，推动优质教育资源在线辐射农村和边远地区薄弱学校，加快发展适合不同学生的信息化自主学习方式，满足个性化发展需求。提高民族地区教育质量和水平，加大国家通用语言文字推广力度。深化民族团结进步教育。
　　5．逐步推进学前教育全面普及。继续实施学前教育行动计划，重点补齐人口集中流入地、农村地区、欠发达地区、民族地区以及城市薄弱地区的普惠性资源短板，基本实现学前教育公共服务体系全覆盖。加强学前幼儿普通话教育，推进学前学会普通话。严格落实城镇小区配套幼儿园政策，鼓励国有企事业单位、街道、村集体举办公办幼儿园。健全普惠性学前教育成本分担机制，建立公办园收费标准动态调整机制，加强非营利性民办园收费监管，遏制过度逐利行为。注重科学保教，建立健全幼儿园保教质量监测体系，坚决克服和纠正“小学化”倾向，全面提升保教质量。
　　6．推进城乡义务教育一体化发展。科学规划布局城乡学校建设，落实新建居住区配套建设学校规定，合理有序扩大城镇学校学位供给，解决城镇大班额问题。全面加强乡村小规模学校和乡镇寄宿制学校建设，进一步改善农村学校办学条件，加强乡村教师队伍建设，提升农村义务教育质量，推进义务教育优质均衡发展。健全精准控辍保学长效机制，提高义务教育巩固水平。保障女童平等接受义务教育。推进义务教育学校免试就近入学全覆盖，全面实行义务教育公办民办学校同步招生，支持和规范民办义务教育发展。
　　7．进一步普及高中阶段教育。加快普通高中育人方式改革，推动高中阶段学校多样化有特色发展，满足学生个性化、多样化发展需要。推进中等职业教育和普通高中教育协调发展，建立中等职业教育和普通高中统一招生平台，深化职普融通。大力发展中等职业教育，建设一批优秀中职学校和优质专业，放宽中职招生地域限制。完善高中阶段学生资助政策。
　　8．保障特殊儿童群体受教育权利。完善特殊教育保障机制，推进适龄残疾儿童教育全覆盖，提高特殊教育质量。坚持以普通学校随班就读为主体，以特殊教育学校为骨干，以送教上门和远程教育为补充，全面推进融合教育。大力发展残疾儿童学前教育，进一步提高残疾儿童义务教育巩固水平，加快发展以职业教育为重点的残疾人高中阶段教育。推进孤独症儿童教育工作。保障农业转移人口随迁子女平等享有基本公共教育服务。加强家庭经济困难学生精准资助，完善奖学金、助学金和助学贷款政策。加强对留守儿童和困境儿童的法治教育、安全教育和心理健康教育，优先满足留守儿童寄宿需求。在特殊教育学校大力推广国家通用手语和国家通用盲文。
　　9．提高儿童科学素质。实施未成年人科学素质提升行动。将弘扬科学精神贯穿教育全过程，开展学前科学启蒙教育，提高学校科学教育质量，完善课程标准和课程体系，丰富课程资源，激发学生求知欲和想象力，培养儿童的创新精神和实践能力，鼓励有创新潜质的学生个性化发展。加强社会协同，注重利用科技馆、儿童中心、青少年宫、博物馆等校外场所开展校外科学学习和实践活动。广泛开展社区科普活动。加强专兼职科学教师和科技辅导员队伍建设。完善科学教育质量和未成年人科学素质监测评估。
　　10．建立健全科学的教育评价制度体系。树立科学的教育质量观，建立健全以发展素质教育为导向的科学评价体系。落实县域义务教育质量、学校办学质量和学生发展质量评价标准。针对不同主体和不同学段、不同类型教育特点，改进结果评价，强化过程评价，探索增值评价，健全综合评价，克服唯分数、唯升学倾向。完善初高中学生学业水平考试和综合素质评价制度。高中阶段学校实行基于初中学业水平考试成绩、结合综合素质评价的招生录取模式，落实优质普通高中招生指标分配到初中政策，健全分类考试、综合评价、多元录取的高校招生机制。
　　11．加强教师队伍建设。完善师德师风建设长效机制，引导教师自觉践行社会主义核心价值观，提升职业道德修养，坚守教书育人职责。完善教师资格准入制度，着力解决教师结构性、阶段性、区域性短缺问题。加强教师进修培训，提高基本功和专业能力。弘扬尊师重教的社会风尚。培养造就一支有理想信念、有道德情操、有扎实学识、有仁爱之心的高素质专业化创新型教师队伍。
　　12．开展民主、文明、和谐、平等的友好型学校建设。加强校风、教风、学风建设，开展文明校园创建，构建尊师爱生的师生关系和团结友爱的同学关系。保障学生参与学校事务的权利。美化校园环境，优化学生学习、生活条件，推进校园无障碍环境建设和改造，为学生提供安全饮用水、卫生厕所和适合身高的课桌椅，改善学校用餐和学生寄宿条件。培育和丰富积极向上、健康文明的校园文化。
　　13．坚持学校教育与家庭教育、社会教育相结合。加强家园、家校协作，推动教师家访制度化、常态化。加强中小学、幼儿园、社区家长学校、家长委员会建设，普及家庭教育知识，推广家庭教育经验。统筹社会教育各类场地、设施和队伍等资源，丰富校外教育内容和形式，鼓励儿童积极参与科技、文化、体育、艺术、劳动等实践活动，参与日常生活劳动、生产劳动和服务性劳动，帮助学生深入了解国情、社情、民情。增强校外教育公益性。加强校外教育理论研究。规范校外培训，切实减轻学生课外培训负担，严格监管面向低龄儿童的校外网络教育培训。充分发挥中小学校课后服务主渠道作用，完善中小学课后服务保障机制和措施，课后服务结束时间原则上不早于当地正常下班时间。发挥共青团、少先队、妇联、科协、关工委等组织的育人作用，形成学校、家庭、社会协同育人合力。
　　（四）儿童与福利。
　　主要目标：
　　1．提升儿童福利水平，基本建成与经济社会发展水平相适应的适度普惠型儿童福利制度体系。
　　2．面向儿童的基本公共服务均等化水平明显提高，城乡、区域和不同群体儿童的公共服务需求得到公平满足。
　　3．巩固提高基本医疗保障水平，保障儿童基本医疗权益。
　　4．构建连续完整的儿童营养改善项目支持体系。
　　5．加快普惠托育服务体系建设，托育机构和托位数量持续增加。
　　6．孤儿、事实无人抚养儿童、残疾儿童、流浪儿童生存、发展和安全权益得到有效保障。
　　7．留守儿童关爱服务体系不断完善，流动儿童服务机制更加健全。
　　8．城乡社区儿童之家覆盖率进一步巩固提高，服务能力持续提升。
　　9．监测预防、强制报告、应急处置、评估帮扶、监护干预“五位一体”的基层儿童保护机制有效运行。县级以上人民政府开通并有效运行全国统一的儿童保护热线。
　　10．基层儿童福利工作阵地和队伍建设进一步加强。
　　11．为儿童服务的社会组织和儿童社会工作专业队伍明显壮大。
　　策略措施：
　　1．完善儿童福利保障和救助制度体系。积极推进儿童福利立法。逐步建成与国家经济社会发展水平相适应、与相关福利制度相衔接的适度普惠型儿童福利制度体系。完善困境儿童分类保障政策，加大困境儿童保障力度。完善儿童福利政策，合理提高儿童福利标准。合理制定低保标准和特困人员救助供养标准并建立动态调整机制，提升儿童生活质量。
　　2．提高面向儿童的公共服务供给水平。提高基本公共服务均等化和可及性水平，将儿童教育、医疗卫生、福利保障事项优先纳入基本公共服务清单，提高服务智慧化水平。完善面向儿童的基本公共服务标准体系，推动基本公共服务向欠发达地区、薄弱环节、特殊儿童群体倾斜。扩大公共服务覆盖面，全面落实儿童乘坐公共交通工具和游览参观票价优惠政策，推进采取年龄标准优先、身高标准补充的儿童票价优待政策。
　　3．做好儿童医疗保障工作。强化基本医疗保险、大病保险、医疗救助三重保障功能，巩固提高儿童参加城乡居民基本医疗保险覆盖率。健全基本医疗保险筹资和待遇调整机制，完善医保目录动态调整机制。做好低收入家庭儿童城乡居民医保参保工作。做好符合条件的家庭经济困难患儿医疗救助。促进各类医疗保障互补衔接，支持商业保险机构开发并推广适宜不同年龄阶段儿童的大病和意外伤害险产品，统筹调动慈善医疗救助力量，支持医疗互助有序发展，合力降低患儿家庭医疗费用负担。
　　4．推进实施儿童营养改善项目。巩固脱贫地区儿童营养改善项目实施成果。稳妥推进农村义务教育学生营养改善计划，完善膳食费用分摊机制。加强3-5岁学龄前儿童营养改善工作，实施学龄前儿童营养改善计划，构建从婴儿期到学龄期连续完整的儿童营养改善项目支持体系。
　　5．发展普惠托育服务体系。将婴幼儿照护服务纳入经济社会发展规划，研究编制托育服务发展专项规划，强化政策引导，综合运用土地、住房、财政、金融、人才等支持政策，扩大托育服务供给。大力发展多种形式的普惠托育服务，推动建设一批承担指导功能的示范托育服务机构和社区托育服务设施，支持有条件的用人单位为职工提供托育服务，鼓励国有企业等主体积极参与各级政府推动的普惠托育服务体系建设，支持和引导社会力量依托社区提供普惠托育服务，鼓励和支持有条件的幼儿园招收2-3岁幼儿，制定家庭托育点管理办法。加大专业人才培养培训力度，依法逐步实行从业人员职业资格准入制度。制定完善托育服务的标准规范，加强综合监管，推动托育服务规范健康发展。
　　6．加强孤儿和事实无人抚养儿童保障。落实孤儿和事实无人抚养儿童保障政策，明确保障对象，规范认定流程，合理确定保障标准。畅通亲属抚养、家庭寄养、机构养育和依法收养孤儿安置渠道。落实社会散居孤儿、事实无人抚养儿童监护责任。完善儿童收养相关法规政策，引导鼓励国内家庭收养病残儿童。健全收养评估制度，建立收养状况回访监督制度，加强收养登记信息化建设。推动收养工作高质量发展。
　　7．落实残疾儿童康复救助制度。完善儿童残疾筛查、诊断、治疗、康复一体化工作机制，建立残疾报告和信息共享制度。提高残疾儿童康复服务覆盖率，为有需求的残疾儿童提供康复医疗、康复辅助器具、康复训练等基本康复服务，促进康复辅助器具提质升级。完善残疾儿童康复服务标准，增强残疾儿童康复服务供给能力，规范残疾儿童康复机构管理。支持儿童福利机构面向社会残疾儿童开展替代照料、养育教育辅导、康复训练等服务。
　　8．加强流浪儿童救助保护工作。落实流浪儿童街面巡查和转介处置职责，依法依规为流浪儿童提供生活照料、身份查询、接送返回等服务。流出地县级政府建立源头治理和回归稳固机制，落实流浪儿童相关社会保障和义务教育等政策，教育督促流浪儿童父母或其他监护人履行抚养义务。依法严厉打击遗弃、虐待未成年人违法犯罪行为。
　　9．加强留守儿童关爱保护。进一步完善留守儿童关爱保护工作体系。强化家庭监护主体责任，提高监护能力。强化县、乡级政府属地责任，落实关爱帮扶政策措施。常态化开展寒暑假特别关爱行动，充分发挥群团组织以及社会组织、社会工作者、志愿者等作用，加强对留守儿童心理、情感、行为和安全自护的指导服务。积极倡导企业履行社会责任，为务工人员加强与留守未成年子女的联系沟通提供支持。落实支持农民工返乡就业创业相关政策措施，从源头上减少留守儿童现象。
　　10．完善流动儿童服务机制。深化户籍制度改革，健全以居住证为载体、与居住年限等条件挂钩的基本公共服务提供机制，推进城镇常住人口基本公共服务均等化，保障儿童平等享有教育、医疗卫生等基本公共服务。健全以社区为依托、面向流动儿童家庭的管理和服务网络，提升专业服务能力，促进流动儿童及其家庭融入社区。
　　11．提高儿童之家建设、管理和服务水平。健全政府主导、部门统筹、多方参与、共同建设儿童之家的工作格局，巩固提高儿童之家覆盖率。完善儿童之家建设标准、工作制度和管理规范，配备专兼职工作人员，发挥社区儿童主任和妇联执委作用，提升管理和使用效能。保证儿童之家服务时长，拓展服务内容，确保服务安全，通过购买服务、项目合作等方式引入有资质的相关社会组织为儿童提供专业化、精细化服务，充分发挥儿童之家在基层社会治理和儿童保护中的作用。
　　12．建立健全基层儿童保护机制。完善县（市、区、旗）、乡镇（街道）、村（社区）三级儿童保护机制。督促学校、幼儿园、托育机构、医疗机构、儿童福利机构、未成年人救助保护机构、村（居）民委员会等主体强化主动报告意识，履行困境儿童和受暴力伤害儿童强制报告义务。开通全国统一的儿童保护热线，及时受理、转介侵犯儿童合法权益的投诉、举报，探索完善接报、评估、处置、帮扶等一体化工作流程，明确相关部门工作职责和协作程序，形成“一门受理、协同办理”的工作机制。
　　13．提升未成年人救助保护机构、儿童福利机构和基层儿童工作队伍服务能力。推进承担集中养育职能的儿童福利机构优化提质，推进儿童养育、康复、教育、医疗、社会工作一体化发展。整合县级儿童福利机构和未成年人救助保护相关机构职能，为临时监护情形未成年人、社会散居孤儿、留守儿童和困境儿童等提供服务。加强未成年人救助保护中心建设，制定完善未成年人救助保护机构工作标准。进一步落实未成年人救助保护机构、儿童督导员、儿童主任的工作职责，加大儿童督导员、儿童主任等基层儿童工作者培训力度，提高服务能力。
　　14．支持引导社会力量参与儿童保护和服务工作。通过政府委托、项目合作、重点推介、孵化扶持等方式，积极培育为儿童服务的社会组织和志愿服务组织。将更多符合条件的儿童保护和服务事项纳入政府购买服务指导性目录，积极引导为儿童服务的社会组织面向城乡社区、家庭和学校提供服务。加强儿童社会工作专业队伍建设，提高服务技能水平。引导社会资源向欠发达地区倾斜，扶持欠发达地区为儿童服务的社会组织发展。
　　（五）儿童与家庭。
　　主要目标：
　　1．发挥家庭立德树人第一所学校作用，培养儿童的好思想、好品行、好习惯。
　　2．尊重儿童主体地位，保障儿童平等参与自身和家庭事务的权利。
　　3．教育引导父母或其他监护人落实抚养、教育、保护责任，树立科学育儿理念，掌握运用科学育儿方法。
　　4．培养儿童成为好家风的践行者和传承者。
　　5．增强亲子互动，建立平等和谐的亲子关系。
　　6．覆盖城乡的家庭教育指导服务体系基本建成，指导服务能力进一步提升。95%的城市社区和85%的农村社区（村）建立家长学校或家庭教育指导服务站点。
　　7．支持家庭生育养育教育的法律法规政策体系基本形成。
　　8．提升家庭领域理论和实践研究水平，促进成果转化应用。
　　策略措施：
　　1．将立德树人落实到家庭教育各方面。父母或其他监护人应将立德树人作为家庭教育的首要任务，将思想品德教育融入日常生活，帮助儿童开拓视野、认识社会，通过身边人、身边事，培养儿童的好思想、好品德、好习惯，引导儿童树立正确的世界观、人生观、价值观。教育引导儿童践行社会主义核心价值观，学习中华民族优秀传统文化，厚植爱党爱祖国爱社会主义情怀。增强法治意识和社会责任感，从小学会做人、学会做事、学会学习，扣好人生第一粒扣子。
　　2．尊重儿童主体地位和权利。引导父母或其他监护人以儿童为本，尊重儿童的身心发展规律和特点，保障儿童休息、锻炼、闲暇和娱乐的权利，合理安排儿童的学习和生活，增加体育锻炼、劳动实践、休息娱乐、社会实践、同伴交往、亲子活动等时间。尊重儿童的知情权、参与权，重视听取并采纳儿童的合理意见。教育引导儿童增强家庭和社会责任意识，鼓励儿童自主选择、自我管理、自我服务，参与力所能及的家务劳动，培养劳动习惯，提高劳动技能。
　　3．增强监护责任意识和能力。创造良好家庭环境，满足儿童身心发展需要，培养儿童良好行为习惯和健康生活方式，提高安全意识和自救自护能力。加强宣传教育培训，帮助父母或其他监护人学习家庭教育知识，树立科学育儿理念和正确成才观，掌握科学育儿方法，尊重个体差异，因材施教。禁止对儿童殴打、虐待等一切形式的家庭暴力。加强对家庭落实监护责任的支持、监督和干预，根据不同需求为家庭提供分类指导和福利保障。
　　4．用好家风培养熏陶儿童。发挥父母榜样和示范作用，教育引导儿童传承尊老爱幼、男女平等、夫妻和睦、勤俭持家、亲子平等、邻里团结的家庭美德，践行爱国爱家、相亲相爱、向上向善、共建共享的社会主义家庭文明新风尚。广泛开展内容丰富、形式新颖、富有教育意义的好家风宣传弘扬活动，推出系列家风文化服务产品。引领儿童养成文明健康的生活方式和消费模式，杜绝浪费。
　　5．培育良好亲子关系。引导家庭建立有效的亲子沟通方式，加强亲子交流，增加陪伴时间，提高陪伴质量。鼓励支持家庭开展亲子游戏、亲子阅读、亲子运动、亲子出游等活动。指导帮助家庭调适亲子关系，缓解育儿焦虑，化解亲子矛盾。鼓励支持各类教育、科技、文化、体育、娱乐等公益性设施和场所以及城乡社区儿童之家等为开展家庭亲子活动提供条件。加强亲子阅读指导，培养儿童良好阅读习惯。分年龄段推荐优秀儿童书目，完善儿童社区阅读场所和功能，鼓励社区图书室设立亲子阅读区。
　　6．构建覆盖城乡的家庭教育指导服务体系。依托现有机构设立家庭教育指导服务中心，统筹家庭教育指导服务工作，依托家长学校、城乡社区公共服务设施、妇女之家、儿童之家等设立家庭教育指导服务站点。建设家庭教育信息化共享平台，开设网上家长学校和家庭教育指导课程。中小学、幼儿园健全家庭教育指导服务工作制度，将家庭教育指导服务纳入学校工作计划和教师业务培训。社区（村）支持协助家庭教育指导服务站点开展家庭教育指导服务。鼓励支持公共文化服务场所开展家庭教育指导活动，利用多种媒体开展家庭教育知识宣传。
　　7．强化对家庭教育指导服务的支持保障。推进实施家庭教育工作规划，推动家庭教育指导服务普惠享有，并纳入政府购买服务指导性目录，培育家庭教育服务机构。加强对家庭教育服务机构和从业人员的管理，规范家庭教育服务市场。加强家庭教育服务行业自律，研究制定服务质量标准，建立行业认证体系。鼓励机关、企事业单位和社会组织面向本单位职工开展家庭教育指导服务。支持社会工作机构、志愿服务组织和专业工作者依法依规开展家庭教育指导服务。
　　8．完善支持家庭生育养育教育的法律法规政策。推进家庭教育立法及实施。完善三孩生育政策配套措施。促进出生人口性别比趋于正常。提高优生优育服务水平，增加优质普惠托育服务供给，推进教育公平与优质教育资源供给，落实产假制度和生育津贴，探索实施父母育儿假。推动将3岁以下婴幼儿照护服务费用纳入个人所得税专项附加扣除，加强住房等支持政策，减轻生育养育教育负担。将困境儿童及其家庭支持与保障作为家庭支持政策的优先领域。加快完善家政服务标准，提高家庭服务智慧化和数字化水平。鼓励用人单位创办母婴室和托育托管服务设施，实施弹性工时、居家办公等灵活的家庭友好措施。
　　9．加强家庭领域理论和实践研究。充分发挥学术型社会组织作用，鼓励有条件的高校和科研机构开设家庭教育专业和课程，建立家庭领域研究基地，培养壮大家庭领域研究队伍，提升研究水平。坚持问题导向，聚焦家庭建设、家庭教育、家风培树等开展研究，及时推进研究成果转化，为家庭领域相关工作提供理论支撑。
　　（六）儿童与环境。
　　主要目标：
　　1．将儿童优先理念落实到公共政策制定、公共设施建设、公共服务供给各方面，尊重、爱护儿童的社会环境进一步形成。
　　2．提供更多有益于儿童全面发展的高质量精神文化产品。
　　3．保护儿童免受各类传媒不良信息影响。提升儿童媒介素养。
　　4．儿童参与家庭、学校和社会事务的权利得到充分保障。
　　5．建设儿童友好城市和儿童友好社区。
　　6．增加公益性儿童教育、科技、文化、体育、娱乐等校外活动场所，提高利用率和服务质量。
　　7．减少环境污染对儿童的伤害。农村自来水普及率达到90%，稳步提高农村卫生厕所普及率。
　　8．提高儿童生态环境保护意识，帮助养成绿色低碳生活习惯。
　　9．预防和应对突发事件时充分考虑儿童的身心特点，优先满足儿童的特殊需求。
　　10．儿童事务国际交流与合作广泛开展，在促进全球儿童事业发展中的作用进一步彰显。
　　策略措施：
　　1．全面贯彻儿童优先原则。建立和完善促进儿童优先发展的制度体系，提高政府部门和社会公众对儿童权利的认识，增强保障儿童权利的自觉性。在出台法律、制定政策、编制规划、部署工作时优先考虑儿童利益和需求。鼓励企事业单位、各类公共服务机构和社会组织参与儿童发展和权利保护服务。在城乡建设规划和城市改造中提供更多适合儿童的公共设施和活动场所。
　　2．提升面向儿童的公共文化服务水平。制作和传播体现社会主义核心价值观、适合儿童的图书、电影、歌曲、游戏、广播电视节目、动画片等精神文化产品，培育儿童文化品牌。支持儿童参与民族优秀传统文化的保护、传承和创新。探索在网络空间开展儿童思想道德教育的新途径、新方法，增强知识性、趣味性和时代性。支持儿童题材作品参加国家舞台艺术精品创作扶持工程等重大项目和重要展演节庆活动。鼓励社会组织、文化艺术机构为儿童文化艺术活动提供专业指导和场地支持。公共图书馆单设儿童阅览区，公共图书馆盲人阅览区为盲童阅读提供便利，鼓励社区图书室设立儿童图书专区。
　　3．加强新闻出版、文化等领域市场监管和执法。加强对儿童出版物的审读、鉴定和处置，深化“扫黄打非”工作，清除淫秽色情低俗、暴力恐怖迷信等有害出版物及信息。清理校园周边非法销售出版物和涉及低俗内容的儿童文化用品、玩具。严格网络出版、文化市场管理与执法，及时整治网络游戏、视频、直播、社交、学习类移动应用软件传播危害未成年人身心健康的有害信息。严格管控诱导未成年人无底线追星、拜金炫富等存在价值导向问题的不良信息和行为。加强互联网营业场所和娱乐场所执法，查处违规接纳未成年人、提供含有禁止内容的曲目和游戏游艺设备等违规行为。落实互联网企业主体责任，在产品开发、内容审核、用户管理、保护措施、举报处置等环节完善治理手段。
　　4．规范与儿童相关的广告和商业性活动。规范与儿童有关的产品（服务）广告播出。在针对儿童的大众传播媒介上不得发布医疗、药品、医疗器械、保健食品、化妆品、酒类、美容广告，以及不利于儿童身心健康的网络游戏广告。禁止在大众传播媒介、公共场所发布声称全部或部分替代母乳的婴儿乳制品、饮料和其他食品广告。加大对相关虚假违法广告案件的查处力度。规范和限制安排儿童参加商业性展演活动。
　　5．加强儿童媒介素养教育。保障儿童利用和参与媒介的权利。丰富儿童数字生活体验，提高数字生活质量。通过学校、幼儿园、家庭和社会等多种渠道，提升儿童及其监护人媒介素养，加强对不同年龄阶段儿童使用网络的分类教育指导，帮助儿童掌握网络基本知识技能，提高学习交流的能力，养成良好用网习惯，引导儿童抵制网络不文明行为，增强信息识别和网上自我保护能力，防止沉迷网络。为欠发达地区儿童、残疾儿童、困境儿童安全合理参与网络提供条件。
　　6．保障儿童参与和表达的权利。尊重儿童参与自身和家庭事务的权利，培养儿童参与意识和能力。涉及儿童的法规政策制定、实施和评估以及重大事项决策，听取儿童意见。将儿童参与纳入学校、校外教育机构、社区工作计划。支持共青团、少先队、妇联等组织开展社会实践及体验活动。加强学校班委会和学生会建设，畅通学生参与学校事务的渠道。广泛开展儿童参与的宣传、教育和培训活动。
　　7．开展儿童友好城市和儿童友好社区创建工作。鼓励创建社会政策友好、公共服务友好、权利保障友好、成长空间友好、发展环境友好的中国特色儿童友好城市。建立多部门合作工作机制，制定适合我国国情的儿童友好城市和儿童友好社区标准体系和建设指南，建设一批国家儿童友好城市。积极参与国际儿童友好城市建设交流活动。
　　8．加大儿童校外活动场所建设和管理力度。将儿童活动场所建设纳入地方经济社会发展规划，加强各类爱国主义教育基地、党史国史教育基地、民族团结进步教育基地、科普教育基地、中小学生研学实践教育基地等的建设，加大对农村地区儿童活动场所建设和运行的扶持力度，推进儿童活动场所无障碍建设和改造。规范儿童校外活动场所管理，各类公益性教育、科技、文化、体育、娱乐场所对儿童免费或优惠开放，根据条件开辟儿童活动专区。在具备条件的校外活动场所普遍建立少先队组织。发挥校外活动场所的育人优势，打造特色鲜明、参与面广的儿童主题活动品牌。
　　9．优化儿童健康成长的自然环境和人居环境。控制和治理大气、水、土壤等环境污染以及工业、生活和农村面源污染，加强水源保护和水质监测。加强铅等重金属污染防治和监测。推进城市饮用水水源地规范化建设。实施农村供水保障工程，提高农村集中供水率、自来水普及率、水质达标率和供水保证率。深入开展爱国卫生运动，持续改善村容村貌和人居环境，分类有序推进农村厕所革命，统筹农村改厕和污水、黑臭水体治理，因地制宜建设污水处理设施。
　　10．创新开展面向儿童的生态文明宣传教育活动。把生态文明教育纳入国民教育体系，融入课堂教学、校园活动、社会实践等环节。推进生态环境科普基地和中小学环境教育社会实践基地建设，在世界环境日、全国低碳日、全国科技活动周等节点开展丰富的儿童环保主题活动，依托自然保护地等建设儿童自然教育保护基地，开展儿童自然教育。培养儿童生态文明意识，树立珍惜资源、保护自然、珍爱生命、与自然和谐相处的观念，自觉养成健康文明、绿色低碳、垃圾分类的良好生活习惯。
　　11．在突发事件预防和应对中加强对儿童的保护。在制定突发事件应急预案时统筹考虑儿童的特殊需求。制定儿童防护用品标准，应急处置期间，优先保证儿童食品、药品、用品供给。学校、幼儿园、托育机构、校外教育机构和社区开展形式多样的安全教育和应急演练活动，提高教职工、儿童及其监护人识别灾害事故风险和应对灾害事故的能力。公共场所发生突发事件时，应优先救护儿童。在灾后恢复与重建阶段，针对儿童特点采取优先救助和康复措施，将灾害事故对儿童的伤害降到最低程度。
　　12．开展促进儿童发展的国际交流与合作。认真履行联合国《儿童权利公约》等国际公约和文件，落实与儿童发展相关的可持续发展目标，扩大多边、双边特别是与共建“一带一路”国家的交流与合作，吸收借鉴国际社会在儿童领域的有益经验，积极宣介促进儿童发展的“中国故事”，在推动构建人类命运共同体、促进全球儿童事业发展中贡献中国智慧、彰显中国担当。
　　（七）儿童与法律保护。
　　主要目标：
　　1．完善保障儿童权益的法律体系。
　　2．加强保障儿童权益的执法工作。
　　3．完善司法保护制度，司法工作体系满足儿童身心发展特殊需要。
　　4．儿童法治素养和自我保护意识进一步提升，社会公众保护儿童的意识和能力进一步提高。
　　5．依法保障儿童的民事权益。
　　6．落实儿童监护制度，保障儿童获得有效监护。
　　7．禁止使用童工，禁止对儿童的经济剥削，严格监管安排儿童参与商业性活动的行为。
　　8．依法严惩性侵害、家庭暴力、拐卖、遗弃等侵犯儿童人身权利的违法犯罪行为。
　　9．依法严惩利用网络侵犯儿童合法权益的违法犯罪行为。
　　10．预防未成年人违法犯罪，对未成年人违法犯罪实行分级干预。降低未成年人犯罪人数占未成年人人口数量的比重。
　　策略措施：
　　1．完善落实保障儿童权益的法律法规。健全保障儿童生存权、发展权、受保护权、参与权的法律法规体系。推动加快家庭教育、学前教育、儿童福利、网络保护等立法进程，适时修订相关法律法规、出台司法解释。增强立法工作的科学性和可操作性。加强[未成年人保护法](https://www.pkulaw.com/chl/883cecd7fe38636dbdfb.html)实施，落实法律监督、司法建议和法治督察制度。加强保障儿童权益的法学理论与实践研究。
　　2．严格保障儿童权益执法。全面落实保障儿童权益主体责任。加大行政执法力度，及时发现和处置监护侵害、家庭暴力、校园及周边安全隐患、食品药品安全隐患等问题。探索建立保障儿童权益多部门综合执法制度，探索建立儿童救助协作制度，强化部门间信息沟通和工作衔接，形成执法、保护、服务合力。
　　3．健全未成年人司法工作体系。公安机关、人民检察院、人民法院和司法行政部门应当确定专门机构或者指定专门人员负责办理涉及未成年人案件。完善未成年人司法保护工作评价考核标准。加强专业化办案与社会化保护配合衔接，加强司法机关与政府部门、人民团体、社会组织和社会工作者等的合作，共同做好未成年人心理干预、社会观护、教育矫治、社区矫正等工作。
　　4．加强对未成年人的特殊司法保护。依法保障涉案未成年人的隐私权、名誉权以及知情权、参与权等诉讼权利。落实未成年人犯罪案件特别程序关于严格限制适用逮捕措施、法律援助、社会调查、心理评估、法定代理人或合适成年人到场、附条件不起诉、不公开审理、犯罪记录封存等规定。增强未成年人社区矫正实施效果。落实涉案未成年人与成年人分别关押、分别管理、分别教育制度。
　　5．依法为儿童提供法律援助和司法救助。依托公共法律服务平台，为儿童提供法律咨询等法律援助服务，推进法律援助标准化、规范化建设，推进专业化儿童法律援助队伍建设。保障符合司法救助条件的儿童获得有针对性的经济救助、身心康复、生活安置、复学就业等多元综合救助。
　　6．加强儿童保护的法治宣传教育。完善学校、家庭、社会共同参与的儿童法治教育工作机制，提高儿童法治素养。扎实推进青少年法治教育实践基地、法治资源教室和网络平台建设，运用法治副校长、以案释法、模拟法庭等多样化方式深入开展法治教育和法治实践活动。提高社会公众的法治意识，推动形成依法保障儿童权益的良好氛围。引导媒体客观、审慎、适度采访和报道涉未成年人案件。
　　7．全面保障儿童的民事权益。依法保障儿童的人身权利、财产权利和其他合法权益。开展涉及儿童权益纠纷调解工作，探索父母婚内分居期间未成年子女权益保护措施，依法保障父母离婚后未成年子女获得探望、抚养、教育、保护的权利。依法保障儿童和胎儿的继承权和受遗赠权。依法保护儿童名誉、隐私和个人信息等人格权。完善支持起诉制度。对食品药品安全、产品质量、烟酒销售、文化宣传、网络传播等领域侵害儿童合法权益的行为，开展公益诉讼工作。
　　8．完善落实监护制度。强化父母或其他监护人履行对未成年子女的抚养、教育和保护职责，依法规范父母或其他监护人委托他人照护未成年子女的行为。督促落实监护责任，禁止早婚早育和早婚辍学行为。加强对监护的监督、指导和帮助，落实强制家庭教育制度。强化村（居）民委员会对父母或其他监护人监护和委托照护的监督责任，村（居）儿童主任切实做好儿童面临监护风险或受到监护侵害情况发现、核实、报告工作。探索建立监护风险及异常状况评估制度。依法纠正和处理监护人侵害儿童权益事件。符合法定情形的儿童由县级以上民政部门代表国家进行监护。确保突发事件情况下无人照料儿童及时获得临时监护。
　　9．严厉查处使用童工等违法犯罪行为。加强对使用童工行为的日常巡视监察和专项执法检查。严格落实儿童参加演出、节目制作等方面国家有关规定。加强对企业、其他经营组织或个人、网络平台等吸纳儿童参与广告拍摄、商业代言、演出、赛事、节目制作、网络直播等的监督管理。严格执行未成年工特殊保护规定，用人单位定期对未成年工进行健康检查，不得安排其从事过重、有毒、有害等危害儿童身心健康的劳动或者危险作业。
　　10．预防和依法严惩性侵害儿童违法犯罪行为。加强儿童预防性侵害教育，提高儿童、家庭、学校、社区识别防范性侵害和发现报告的意识和能力，落实强制报告制度。建立全国统一的性侵害、虐待、拐卖、暴力伤害等违法犯罪人员信息查询系统，完善落实入职查询、从业禁止制度。探索建立性侵害儿童犯罪人员信息公开制度，严格落实外籍教师无犯罪证明备案制度。加强立案和立案监督，完善立案标准和定罪量刑标准。依法严惩对儿童负有特殊职责人员实施的性侵害行为，依法严惩组织、强迫、引诱、容留、介绍未成年人卖淫犯罪。建立未成年被害人“一站式”取证机制，保护未成年被害人免受“二次伤害”。探索制定性侵害儿童案件特殊证据标准。对遭受性侵害或者暴力伤害的未成年被害人及其家庭实施必要的心理干预、经济救助、法律援助、转学安置等保护措施。
　　11．预防和依法严惩对儿童实施家庭暴力的违法犯罪行为。加强反家庭暴力宣传，杜绝针对儿童的家庭暴力以及严重忽视等不利于儿童身心健康的行为。落实强制报告制度，及时受理、调查、立案和转处儿童遭受家庭暴力案件。出台关于[反家庭暴力法](https://www.pkulaw.com/chl/ac57ba1df4413457bdfb.html)的司法解释、指导意见或实施细则，充分运用告诫书、人身安全保护令、撤销监护人资格等措施，加强对施暴人的惩戒和教育。对构成犯罪的施暴人依法追究刑事责任，从严处理重大恶性案件。保护未成年被害人的隐私和安全，及时为未成年被害人及目睹家庭暴力的儿童提供心理疏导、医疗救治和临时庇护。
　　12．严厉打击拐卖儿童和引诱胁迫儿童涉毒、涉黑涉恶等违法犯罪行为。坚持和完善集预防、打击、救助、安置、康复于一体的反拐工作长效机制，继续实施反对拐卖人口行动计划。有效防范和严厉打击借收养名义买卖儿童、利用网络平台实施拐卖人口等违法犯罪行为，加大对出卖亲生子女犯罪的预防和打击力度，完善孕产妇就医生产身份核实机制，完善亲子鉴定意见书和出生医学证明开具制度，落实儿童出生登记制度。妥善安置查找不到亲生父母和自生自卖类案件的被解救儿童。禁止除公安机关以外的任何组织或者个人收集被拐卖儿童、父母和疑似被拐卖人员DNA数据等信息。实施全国青少年毒品预防教育工程，开展对引诱、教唆、欺骗、强迫、容留儿童吸贩毒犯罪专项打击行动。依法严惩胁迫、引诱、教唆儿童参与黑社会性质组织从事违法犯罪活动的行为。
　　13．严厉打击侵犯儿童合法权益的网络违法犯罪行为。加强对网络空间涉及儿童违法犯罪的分析研究，以案释法，提高公众对儿童网络保护的意识和能力。禁止制作、复制、发布、传播或者持有有关未成年人的淫秽色情物品和网络信息。依法严惩利用网络性引诱、性侵害儿童的违法犯罪行为。禁止对儿童实施侮辱、诽谤、威胁或者恶意损害形象等网络欺凌行为。严厉打击利用网络诱骗儿童参与赌博以及敲诈勒索、实施金融诈骗等违法犯罪行为。
　　14．有效预防未成年人违法犯罪。加强对未成年人的法治和预防犯罪教育。落实未成年人违法犯罪分级干预制度，依法采取教育矫治措施，及时发现、制止、管教未成年人不良行为。及时制止、处理未成年人严重不良行为和未达刑事责任年龄未成年人严重危害他人及社会的行为。完善专门学校入学程序、学生和学籍管理、转回普通学校等制度。对涉罪未成年人坚持依法惩戒与精准帮教相结合，增强教育矫治效果，预防重新犯罪。保障涉罪未成年人免受歧视，依法实现在复学、升学、就业等方面的同等权利。强化预防未成年人犯罪工作的基层基础。

　　**三、**组织实施
　　（一）坚持党的全面领导。坚持以习近平新时代中国特色社会主义思想为指导，坚持以人民为中心的发展思想，坚持走中国特色社会主义儿童发展道路，把党的领导贯穿于纲要组织实施全过程。贯彻党中央关于儿童事业发展的决策部署，在统筹推进“五位一体”总体布局、协调推进“四个全面”战略布局中推进纲要实施。
　　（二）落实纲要实施责任。完善落实党委领导、政府主责、妇儿工委协调、多部门合作、全社会参与的纲要实施工作机制。国务院及地方各级人民政府负责纲要实施工作，各级妇儿工委负责组织、协调、指导、督促工作，各级妇儿工委办公室负责具体工作。有关部门、相关机构和人民团体结合职责，承担纲要相关目标任务落实工作。在出台法律、制定政策、编制规划、部署工作时贯彻落实儿童优先原则，切实保障儿童权益，促进儿童优先发展。
　　（三）加强纲要与国民经济和社会发展规划的衔接。在经济社会发展总体规划及相关专项规划中贯彻落实儿童优先原则，将纲要实施以及儿童发展纳入经济社会发展总体规划及相关专项规划，结合经济社会发展总体规划部署要求推进纲要实施，实现儿童事业发展与经济社会发展同步规划、同步部署、同步推进、同步落实。
　　（四）制定地方儿童发展规划和部门实施方案。省级人民政府依据本纲要，结合实际制定本级儿童发展规划。市、县级人民政府依据本纲要以及上一级儿童发展规划，结合实际制定本级儿童发展规划。省、市、县级规划颁布后1个月内报送上一级妇儿工委办公室。中央及地方承担纲要（规划）目标任务的有关部门、相关机构和人民团体结合职责，按照任务分工，制定实施方案并报送同级妇儿工委办公室。
　　（五）完善实施纲要的工作制度机制。健全目标管理责任制，将纲要实施纳入政府议事日程和考核内容，将纲要目标分解到责任单位并纳入目标管理和考核内容。健全督导检查制度，定期对纲要实施情况开展督查。健全报告制度，责任单位每年向同级妇儿工委报告纲要实施情况和下一年工作安排，下级妇儿工委每年向上一级妇儿工委报告本地区规划实施情况和下一年工作安排。健全议事协调制度，定期召开妇女儿童工作会议和妇儿工委全体会议、联络员会议等，总结交流情况，研究解决问题，部署工作任务。健全纲要实施示范制度，充分发挥示范单位以点带面、示范带动作用。健全表彰制度，对实施纲要先进集体和先进个人按照有关规定进行表彰。
　　（六）加强儿童发展经费支持。各级人民政府将实施纲要所需工作经费纳入财政预算，实现儿童事业和经济社会同步发展。重点支持革命老区、民族地区、边疆地区、欠发达地区儿童发展，支持特殊困难儿童群体发展。动员社会力量，多渠道筹集资源，共同发展儿童事业。
　　（七）坚持和创新实施纲要的有效做法。贯彻新发展理念，坚持问题导向、目标导向，构建促进儿童发展的法律法规政策体系，完善儿童权益保障机制，实施促进儿童发展的民生项目。通过分类指导、示范先行，总结推广好做法好经验。通过政府购买服务等方式，发挥社会力量推动纲要实施的作用。开展国际交流合作，交流互鉴经验做法，讲好中国儿童发展故事。
　　（八）加强纲要实施能力建设。将习近平总书记关于儿童和儿童工作的重要论述以及儿童优先原则有关内容、相关法律法规政策纳入各级干部学习内容，将实施纲要所需知识纳入培训计划，举办多层次、多形式培训，增强政府有关部门、相关机构和人员实施纲要的责任意识和能力。以政治建设为统领，加强各级妇儿工委及其办公室能力建设，促进机构职能优化高效，为更好履职尽责提供必要的人力物力财力支持，为纲要实施提供组织保障。
　　（九）加大纲要宣传力度。大力宣传习近平总书记关于儿童和儿童工作的重要论述，宣传在党的坚强领导下儿童事业发展的成就，宣传儿童优先原则和保障儿童权益、促进儿童发展的法律法规政策，宣传纲要内容和纲要实施的经验、成效，努力营造关爱儿童、有利于儿童发展的社会氛围。
　　（十）加强儿童发展调查研究。充分发挥各级妇儿工委及其办公室作用，加强儿童工作智库建设，依托高校、研究机构、社会组织等建设儿童发展研究基地，培育专业研究力量，广泛深入开展理论与实践研究，为制定完善相关法律法规政策提供参考。
　　（十一）鼓励社会各界广泛参与纲要实施。健全学校、家庭、社会“三位一体”教育网络，鼓励企事业单位、社会组织、慈善机构和公益人士参与保障儿童权益、促进儿童发展等工作。鼓励儿童参与纲要实施，提高儿童在参与纲要实施中实现自身全面发展的意识和能力。

　　**四、**监测评估
　　（一）加强监测评估制度建设。对纲要实施情况进行年度监测、中期评估、终期评估。落实并逐步完善儿童统计监测方案。各级统计部门牵头组织开展年度监测，各级妇儿工委成员单位、有关部门、相关机构向同级统计部门报送年度监测数据，及时收集、分析反映儿童发展状况的相关数据和信息。各级妇儿工委组织开展中期和终期评估，各级妇儿工委成员单位、有关部门、相关机构向同级妇儿工委提交中期和终期评估报告。通过评估，了解掌握纲要实施进展和儿童发展状况，系统分析评价纲要目标任务完成情况，评判纲要策略措施的实施效果，总结经验做法，找出突出问题，预测发展趋势，提出对策建议。监测评估工作所需经费纳入财政预算。
　　（二）加强监测评估工作组织领导。各级妇儿工委设立监测评估领导小组，由同级妇儿工委及相关部门负责同志组成，负责监测评估工作的组织领导、监测评估方案的审批、监测评估报告的审核等。领导小组下设监测组和评估组。
　　监测组由各级统计部门牵头，相关部门负责纲要实施情况统计监测的人员参加，负责监测工作的组织、指导和培训，制定监测方案和指标体系，收集、分析数据信息，向同级妇儿工委提交年度、中期和终期监测报告，编辑出版年度妇女儿童统计资料等。监测组成员负责统筹协调本部门纲要实施监测、分析、数据上报、分性别分年龄指标完善等工作。
　　评估组由各级妇儿工委办公室牵头，相关部门负责纲要实施的人员参加，负责评估工作的组织、指导和培训，制定评估方案，组织开展评估工作，向同级妇儿工委提交中期和终期评估报告。评估组成员负责统筹协调本部门纲要实施自我评估工作，参加妇儿工委组织的评估工作。支持评估组相关部门就儿童保护与发展中的突出问题开展专项调查、评估，结果可供中期和终期评估参考。
　　（三）加强儿童发展统计监测。规范完善儿童发展统计监测指标体系，根据需要调整扩充儿童发展统计指标，推动纳入国家和部门常规统计以及统计调查制度，加强部门分年龄统计工作，推进儿童发展统计监测制度化建设。国家、省、市三级建立完善儿童发展统计监测数据库，支持县级儿童发展统计监测数据库建设。鼓励支持相关部门对儿童发展缺项数据开展专项统计调查。
　　（四）提升监测评估工作能力和水平。加强监测评估工作培训和部门协作，规范监测数据收集渠道、报送方式，提高数据质量。运用互联网和大数据等，丰富儿童发展和分年龄统计信息。科学设计监测评估方案和方法，探索开展第三方评估。提升监测评估工作科学化、标准化、专业化水平。
　　（五）有效利用监测评估成果。发挥监测评估结果服务决策的作用，定期向同级人民政府及相关部门报送监测评估情况，为决策提供依据。建立监测评估报告交流、反馈和发布机制。加强对监测评估结果的研判和运用，对预计完成困难、波动较大的监测指标及时预警，对评估发现的突出问题和短板弱项及时提出对策建议。运用监测评估结果指导下一阶段纲要实施，实现纲要实施的常态化监测、动态化预警、精准化干预、高质量推进。

© 北大法宝：（[www.pkulaw.com](https://www.pkulaw.com/)) 专业提供法律信息、法学知识和法律软件领域各类解决方案。北大法宝为您提供丰富的参考资料，正式引用法规条文时请与标准文本核对。
欢迎查看所有[产品和服务](https://www.pkulaw.com/helps/2.html)。法宝快讯：[如何快速找到您需要的检索结果？](https://www.pkulaw.com/helps/70.html)[法宝](https://www.pkulaw.com/helps/69.html)V6有何新特色？

扫描二维码阅读原文

**原文链接：**<https://www.pkulaw.com/chl/661491589bbbf5a2bdfb.html>
